# Direct Observation of Topologically Enhanced Interfacial Miscibility in Ring–Linear Polymers

**DOI:** 10.1021/acscentsci.6c00828

**Published:** 2026-09-03

**Authors:** Seong Eun Kim, Dogyun Kim, Se Hwa Park, Tae Yeon Kong, June Hyuk Lee, Kyoung Taek Kim, So Youn Kim

**Affiliations:** 1 Department of Chemical and Biological Engineering, Institute of Chemical Processes, 26725 Seoul National University, Seoul 08826, Republic of Korea; 2 Department of Chemistry, College of Natural Science, Seoul National University, Seoul 08826, Republic of Korea; 3 Neutron Science Division, 65405 Korea Atomic Energy Research Institute, Daejeon 34057, Republic of Korea

## Abstract

Cyclic polymers, lacking chain ends, exhibit unique behavior when blended with linear analogues. In such cyclic/linear mixtures, cyclic polymers permit chain threading, wherein linear chains penetrate the ring-like structure, increasing entropy and enhancing interfacial diffusion relative to linear/linear or cyclic/cyclic blends. Although theory has long predicted this topological entropy-driven miscibility, experimental evidence has been scarce due to challenges in synthesis and characterization. Here, we provide the first direct experimental validation using neutron reflectivity on cyclic and linear poly­(lactic acid) (PLA) analogues assembled as bilayers of higher-molecular-weight, isotopically labeled linear PLA in thin films. The results show that topological entropy markedly reduces the effective interaction parameter, χ_eff_, promoting interfacial miscibility. This demonstrates that molecular topology alone can alter macroscopic thermodynamics, offering a fundamental route to control phase behavior in soft condensed matter.

## Graphical abstract

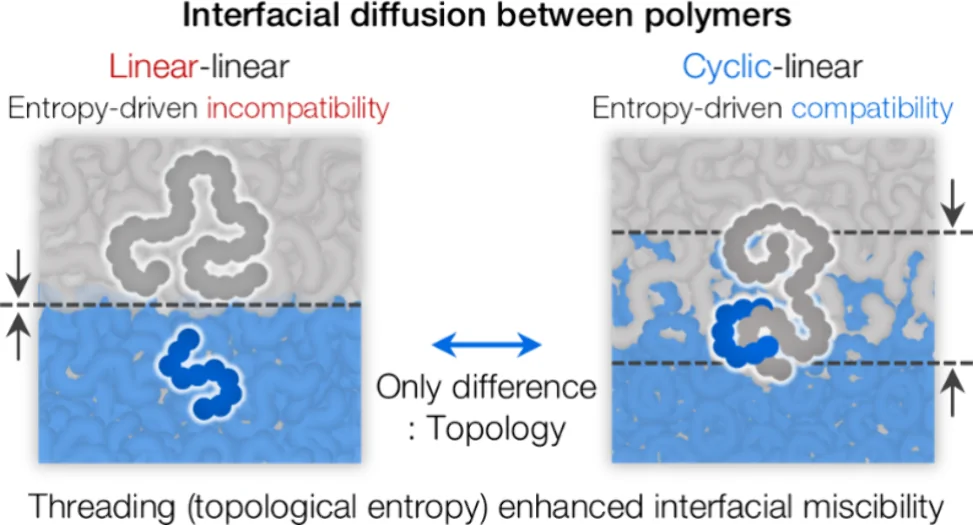

## Introduction

Chemically identical polymers are often expected to mix favorably because the enthalpic contribution to mixing is negligible and translational entropy (S_trans_) favors miscibility.^1,2^ Yet in practice, blends with large molecular-weight disparities often remain immiscible. This apparent contradiction arises from the conformational entropy (S_conf_) penalty associated with interpenetration, which becomes severe when short chains must stretch to mix with much longer ones.^3^ This suggests that reducing the S_conf_ penalty could improve interfacial miscibility, even in chemically identical polymers.

Cyclic polymers differ fundamentally from their linear counterparts. Because they lack free chain ends, they exhibit intrinsically lower S_conf_, arising from the noncatenation constraint, and consequently give rise to unexpected behaviors in dynamics, glass transition, and miscibility.^4–10^ Notably, theoretical and simulation studies have conjectured that cyclic chains exhibit enhanced compatibility or miscibility when blended with linear chains because linear-chain threading through neighboring rings generates an additional topological entropy (S_topo_) gain, which partially offsets the usual entropic penalty of mixing.^11–15^ Figure 1 illustrates the conformational constraints and topological effect found in entropy-driven unmixing/mixing of polymers. Whereas entropy-driven unmixing often occurs in linear–linear blends with different molecular weights, the gain of S_topo_ in linear–cyclic blends instead promotes interfacial mixing. However, until now, this mechanism has remained largely theoretical and unproven experimentally, owing to challenges in synthesizing topologically pure cyclic polymers and isolating entropic effects from other interactions.^16–18^

**Figure 1:**
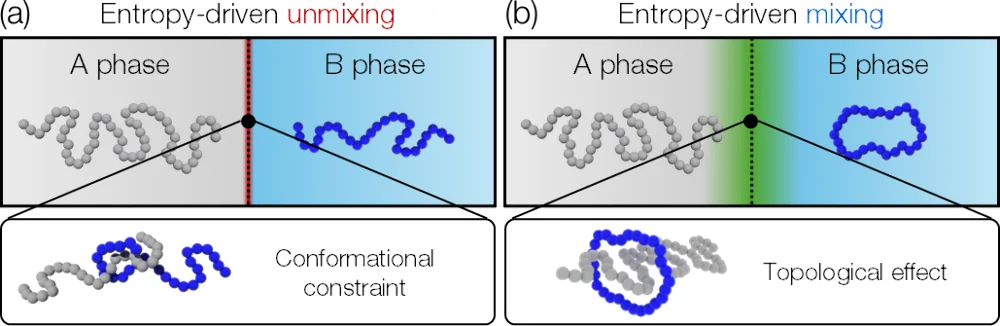
Schematic illustration of entropy-driven (a) unmixing and (b) mixing determined by polymer architecture at the chemically identical polymer interface.

Notably, progress is gradually being made toward overcoming this challenge: cyclic analogues of commodity polymers, such as cyclic polyethylene, polypropylene, and polynorbornene,^19–21^ have begun to become synthetically accessible through ring-expansion strategies. Although precise control over topological purity and molecular weight remains an ongoing pursuit, this emerging progress increasingly links the study of cyclic polymer properties, including interfacial miscibility, to the potential applications of these materials, underscoring the need for a direct experimental understanding of how topology governs interfacial behavior.

Moreover, several studies have reported that cyclic topology can enhance thermodynamic miscibility relative to linear analogues;^22–25^ for example, small-angle neutron scattering on isotopic linear-cyclic blends has revealed an effectively negative linear-cyclic interaction parameter, attributed to topological (entropic) contributions such as reduced threading. These results establish that chain topology alone can modulate miscibility in the bulk.^25^ However, most of these studies address bulk, homogeneously mixed blends. Whether, and to what extent, this topology-driven miscibility manifests at a ring–linear interface remains unexplored. Interfacial mixing involves chain conformations near a sharp concentration gradient, and bulk miscibility alone does not necessarily determine the interfacial behavior between linear and cyclic polymers.

Here, we address this gap by directly probing the interface between cyclic and linear polymers in a bilayer geometry, providing the first quantification of cyclic/linear interfacial miscibility via the gain of S_topo_. Using discrete *linear* or *cyclic* poly­(lactic acid) (PLA), the interfacial diffusion of these polymers into high-molecular-weight deuterated PLA was monitored in thin films via neutron reflectivity (NR). We found that while linear/linear PLA systems remain immiscible although the chemistry of the two layers is identical apart from isotopic labeling, cyclic/linear systems exhibit a broader interdiffusion width. This shift suggests that molecular topology can directly modulate entropy-driven interfacial miscibility, even when polymer chemistry is unchanged. Our results confirm the long-standing prediction, previously advanced by theory and simulation, that S_topo_ gain through chain threading enhances interfacial miscibility in cyclic/linear polymer systems.

## Synthesis and Characterization of h-PLAs

We synthesized linear hydrogenated-PLAs (h-PLAs) having discrete degrees of polymerization (DP) of 128 and 256 via our iterative convergent strategy.^26,27^ The corresponding cyclic PLAs having the same DPs were subsequently prepared through end-group modification followed by intramolecular cyclization reactions (Figure 2(a)). The successful synthesis of discrete linear and cyclic PLAs was confirmed by matrix-assisted laser desorption/ionization time-of-flight (MALDI-TOF) mass spectrometry and gel permeation chromatography (Figure 2(b) and 2(c)). The polymers are denoted as L or C for linear and cyclic ones, respectively, followed by DP. For example, L128 refers to a linear PLA having a DP of 128. A detailed synthesis method is provided in the Supporting Information.

**Figure 2:**
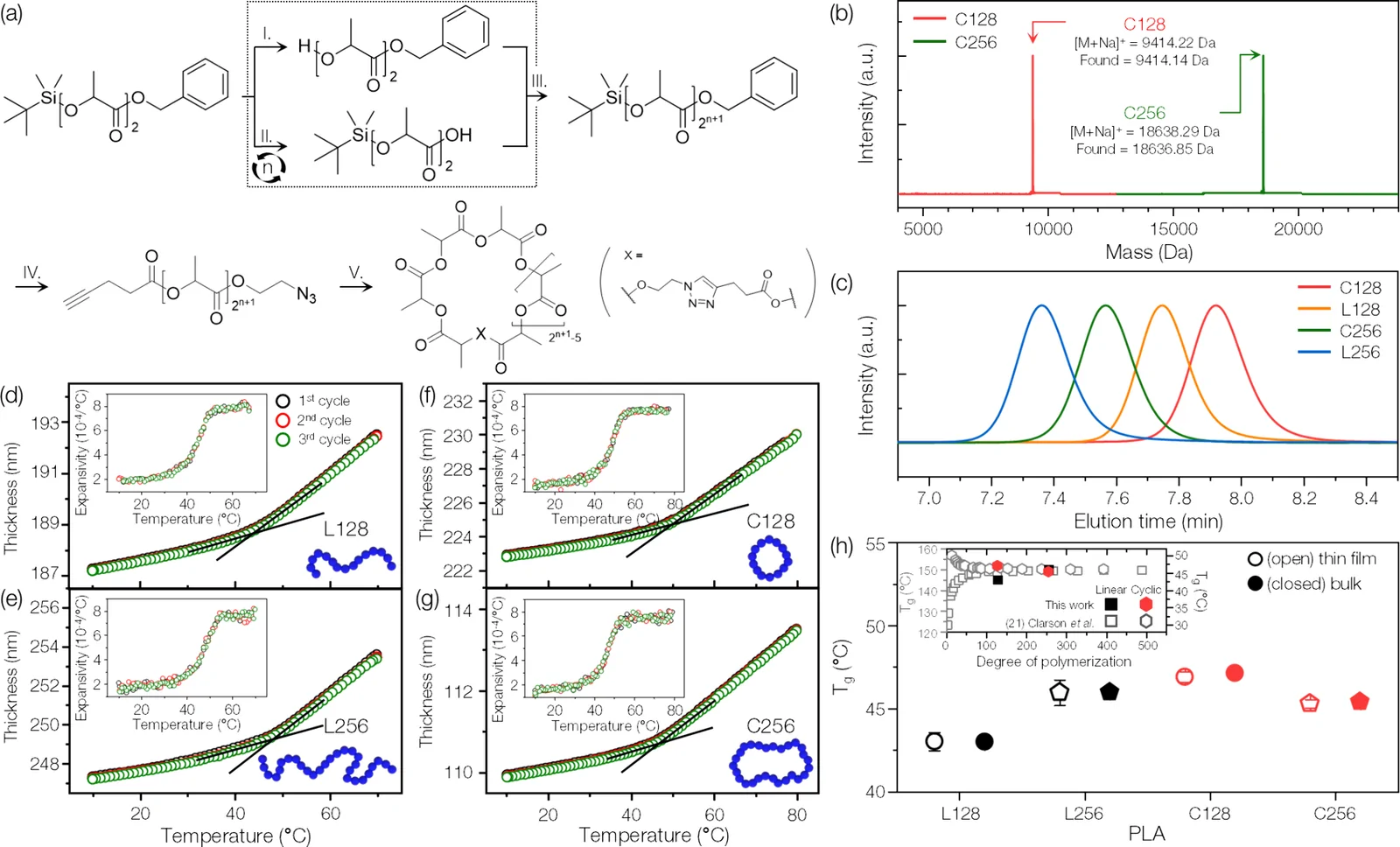
(a) Synthesis of discrete linear and cyclic PLAs (I: Et_3_SiH, Pd/C, EA, rt; II: BF_3_·Et_2_O, DCM, rt; III: *N*-(3-dimethyl­amino­propyl)-*N*′-ethyl­carbodiimide hydrochloride (EDC·HCl), 4-(dimethyl­amino)­pyrid­inium 4-toluene­sulfonate (DPTS), DCM, rt; IV: a Et_3_SiH, Pd/C. b 2-azidoethanol, EDC·HCl, DPTS. c BF_3_·Et_2_O. d 4-pentynoic acid, EDC·HCl, DPTS; V: CuSO_4_·5H_2_O, (+)-sodium l-ascorbate, DMSO, 60 °C. (b) MALDI-TOF mass spectra of C128 and C256. (c) SEC traces of linear and cyclic PLAs. In situ spectroscopic ellipsometry data to measure the *T*_g_ for (d) L128, (e) L256, (f) C128, and (g) C256. Insets are corresponding expansivity data. (h) Extracted *T*_g_ for PLAs in bulk or thin films. Inset is the molecular-weight-dependent *T*_g_ of PLA (red) and poly­(dimethylsiloxanes) (black) reproduced from ref 8. Copyright 1987 American Chemical Society.

The dynamic behavior of the four PLAs is first investigated with glass transition temperature (*T*_g_) measurement. PLA films were prepared with a thickness greater than 100 nm, and the film thickness was monitored using spectroscopic ellipsometry while cooling from 80 to 10 °C at a rate of 3 °C/min. Figure 2(d-g) shows the typical thickness increments with an abrupt change in expansivity (inset), and *T*_g_ was determined from the crossover point between the linear extrapolation of the glassy and liquid regimes. The summarized *T*_g_ results are given in Figure 2(h), exhibiting the *T*_g_ values near 45 °C. Depending on the architecture of the polymers, their molecular weight dependency exhibits opposite trends: *T*_g_ increases with molecular weight for linear analogues, whereas it decreases for cyclic analogues (Figure 2(h), inset). We note that the thin films (50 nm, unfilled symbols) and bulk *T*_g_ (300 nm, filled symbols and Figures S7 and S8) showed the same results. This opposite trend is attributed to the presence of chain ends in linear polymers, which contribute to higher S_conf_. For the linear analogues, freely moving chain ends increase overall S_conf_. As their molecular weight increases, the decreased fraction of chain ends results in a reduction of entropy accompanied by an apparent rise in *T*_g_. In contrast, cyclic analogues lack the chain ends due to the ring closure. Therefore, as their molecular weight increases, the number of configurations increases as well, thereby lowering the *T*_g_. We plotted our results together with previously reported trends with increasing DP as shown in Figure 2(h), inset. While the molecular-weight dependence of *T*_g_ in cyclic polymers remains a matter of ongoing discussion, our results, obtained under contamination-free conditions, are consistent with the entropy-based prediction^8,28^ (see Section F in the Supporting Information for a detailed discussion of this point). Therefore, we expect that chosen molecular weights are effectively low to observe the chain-end effects, enabling direct comparison of the entropy changes due to the ring closure.

## Entropic Control of Interfacial Diffusion

We next investigated the interfacial interdiffusion between d-PLA_43k_ and above four PLAs to examine the limited compatibility between the PLAs arising from the molecular weight disparity. Interdiffusion at the interface of PLA (bottom)/d-PLA_43k_ (top) bilayers was examined using NR at a fixed temperature (Figure 3(a)), with bilayers prepared by the floating technique described in the Supporting Information. Two layers of film interdiffuse one another upon annealing at a temperature higher than *T*_g_, producing the interfacial region of mixing. We compared the thickness of the interfacial region (interdiffusion width) after the equilibration. The choice of the operating temperature and the limited dynamics depending on the temperatures are discussed in the Supporting Information. We note that the use of deuterium rather than hydrogen introduces a small enthalpic contribution, arising from the shorter C–D bond length and correspondingly smaller segmental volume, which yields a positive isotopic interaction parameter (χ_HD_) on the order of χ_HD_ ∼ 10^–3^–10^–4^.^29^ Our system is therefore not strictly zero-enthalpy. Crucially, however, this isotopic contribution is common to both the linear/d-PLA_43k_ and cyclic/d-PLA_43k_ interfaces and therefore cancels when the two topologies are compared, leaving the differential interdiffusion attributable to chain architecture alone.

**Figure 3:**
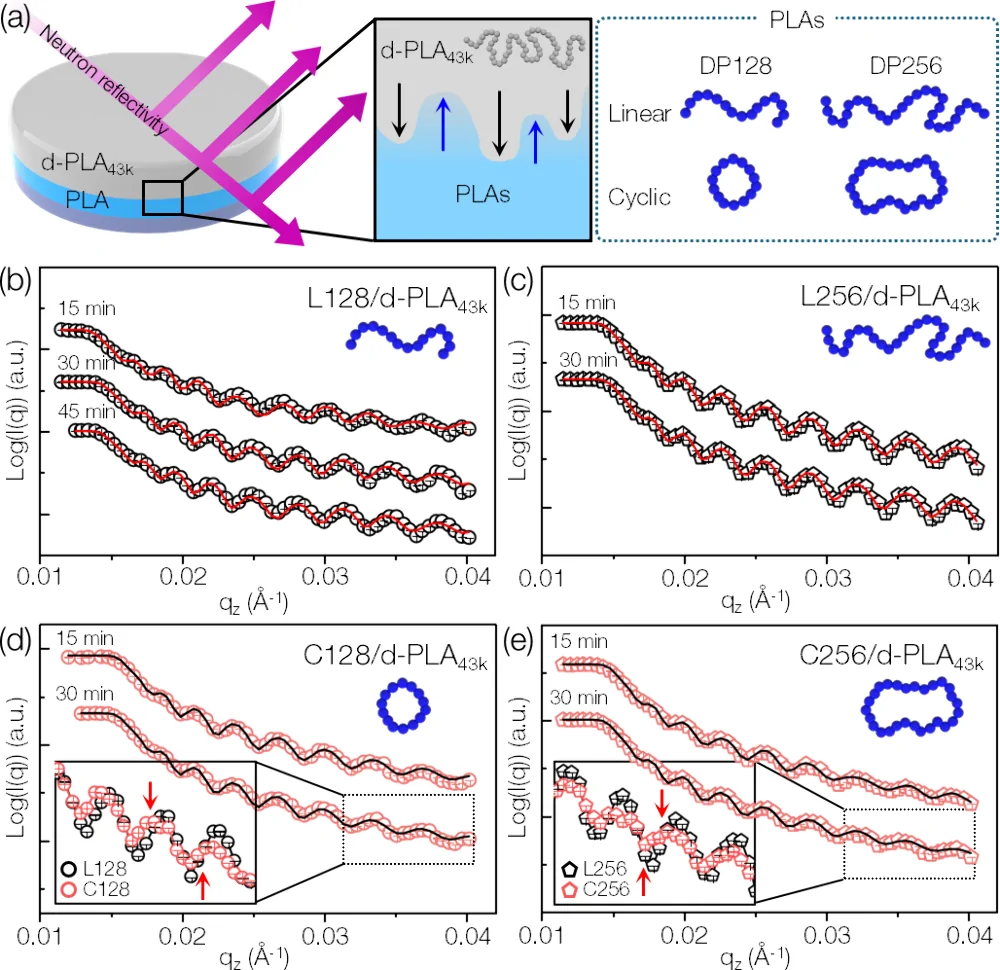
(a) Schematic illustration of the PLA/d-PLA_43k_ bilayer geometry for NR experiments. Reflectivity data of (b) L128/d-PLA_43k_, (c) L256/d-PLA_43k_, (d) C128/d-PLA_43k_, and (e) C256/d-PLA_43k_. Insets are overlapped reflectivity data of linear and cyclic polymers having the same molecular weight.

Figure 3(b-e) shows the obtained intensity spectra from NR experiments and corresponding fitted curves for all four PLA/d-PLA_43k_ bilayers. For the given system, all reflectivity profiles overlapped after holding the samples at 80 °C for 15 min, indicating the system had effectively plateaued under the experimental conditions. The NR profiles for bilayers incorporating linear PLAs (L128 or L256/d-PLA_43k_) exhibit clear Kiessig fringes across the entire q_*z*_ range, indicating well-defined, discrete bilayer structures (Figure 3(b-c)). In contrast, replacing linear h-PLAs with cyclic analogues results in attenuated fringe patterns (Figure 3(d-e)), which suggests a broader interdiffusion region.

To quantify the interdiffusion width, we fitted the reflectivity curves with the two-layer model (different scattering length density (SLD)). The reflectivity curves were well fitted by the two-layer model, and the resulting SLD profiles along the film-thickness direction reveal the presence or absence of an interdiffusion layer. Detailed fitting processes and fitting parameters are described in the Supporting Information. Figure 4 shows that the linear/linear bilayer has no or little interdiffusion layers, whereas the cyclic/linear combination clearly shows the wider range of interdiffusion. The calculated average interdiffusion widths are 0.0 nm for L128/d-PLA_43k_, 1.9 nm for L256/d-PLA_43k_, 4.4 nm for C128/d-PLA_43k_, and 5.0 nm for C256/d-PLA_43k_ (Figure 4(a-d)). We note that the value for L128/d-PLA_43k_ lies below the resolution limit and should therefore be regarded as undetectable value rather than an exact value of zero. In addition, thermally induced capillary waves may also contribute slightly to the extracted interfacial widths, but this does not affect the systematic topology-dependent comparison made under identical measurement conditions.

**Figure 4:**
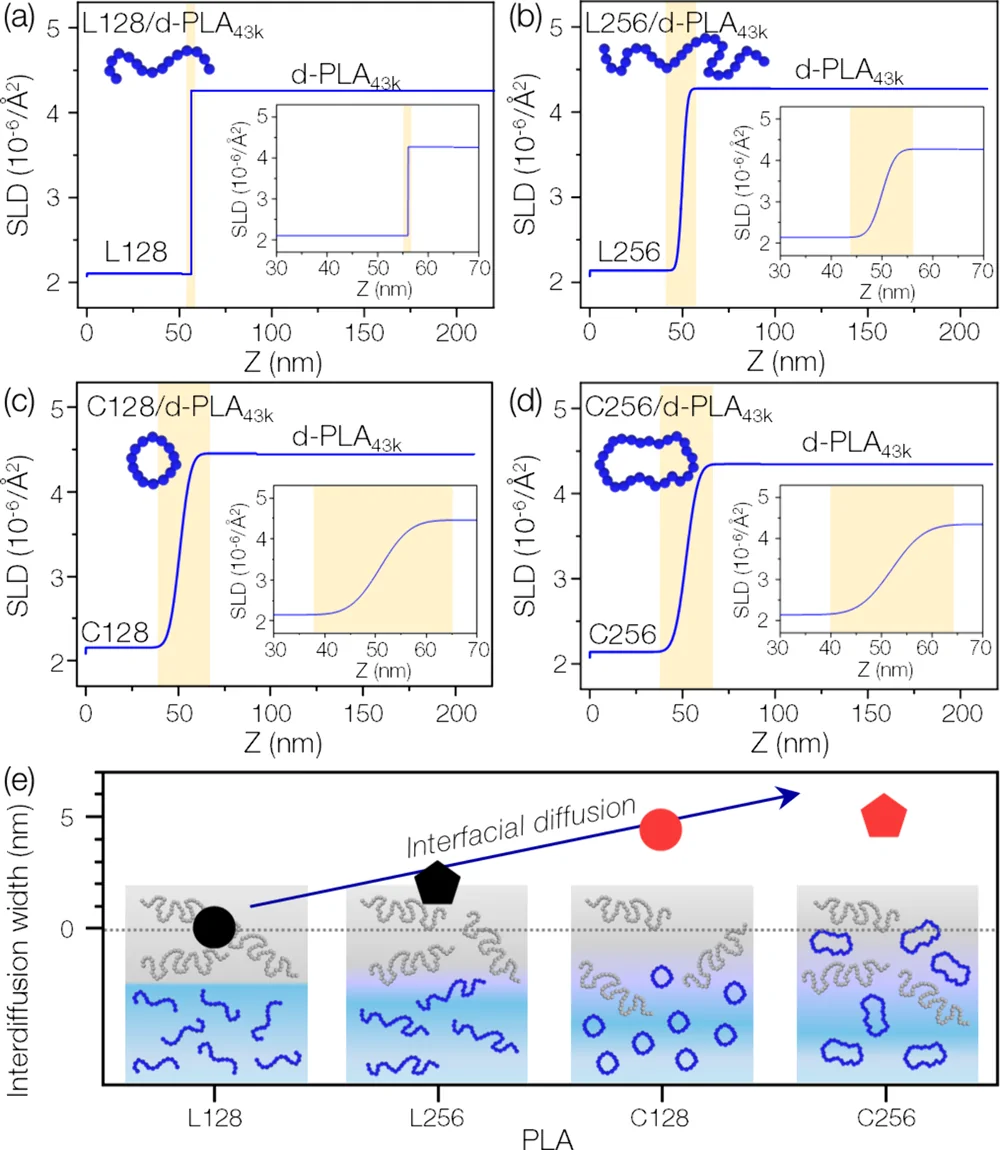
SLD profile obtained from fitting the reflectivity data of (a) L128/d-PLA_43k_, (b) L256/d-PLA_43k_, (c) C128/d-PLA_43k_, and (d) C256/d-PLA_43k_. Insets are zoomed-in profiles near the interdiffusion regime. (e) Interdiffusion width of all bilayer combinations.

These results describe two main trends: (1) higher molecular weight polymers exhibit broader interdiffusion widths regardless of polymer architecture, and (2) cyclic polymers consistently show greater interdiffusion than their linear counterparts across molecular weights. The extracted interdiffusion widths are summarized in Figure 4(e). Notably, no interdiffusion is observed at the L128/d-PLA_43k_ interface, despite the prediction based on classical Gibbs free energy of mixing (ΔG_mix_) as described in eq 1.

1 $$\frac{\Delta G_{\mathrm{mix}}}{RT}=-\frac{\Delta S_{\mathrm{trans}}}{R}+\frac{\Delta H}{RT}=\phi_{1}\frac{\ln\;\phi_{1}}{N_{1}}+\phi_{2}\frac{\ln\;\phi_{2}}{N_{2}}+\chi \phi_{1}\phi_{2}$$

The N is the DP, χ is the interaction parameter, and ϕ is a volume fraction of each component. Because the two polymers are chemically identical, the enthalpic contribution (χ) to mixing is expected to be negligible, while the ΔS_trans_ is positive. Therefore, the conventional ΔG_mix_ predicts a negative and, consequently, interdiffusion. However, the absence of interdiffusion width for L128/d-PLA_43k_ suggests the existence of additional contributors hindering the mixing, such as ΔS_conf_. ΔS_conf_ introduces the effective χ, χ_eff_, and it seems to play a dominant role in suppressing the interfacial miscibility in this system. In addition, cyclic analogues consistently exhibit broader interdiffusion widths than their linear counterparts. Taken together, these results establish that broader interdiffusion can arise solely from differences in polymer architecture, even when polymer chemistry is unchanged.

To ensure that the measured interdiffusion widths reflect thermodynamic rather than kinetic quantities, we confirmed that the interfacial width reaches a plateau and no longer evolves with extended annealing for all bilayers examined (L256/d-PLA_43k_, C128/d-PLA_43k_, and C256/d-PLA_43k_; see Section K in the Supporting Information for detailed information). This time-independence indicates that the reported widths correspond to equilibrium interfacial widths rather than transient, kinetically limited states, and we therefore treat the system as equilibrated in the analysis that follows. Since our samples are isotopically labeled, the relevant interaction parameter is small but positive (χ_H/D_ ∼ 10^–3^–10^–4^), enthalpy does not favor mixing, and interdiffusion is driven entirely by entropy. The equilibrium interfacial width is therefore intrinsically narrow, and complete mixing is not expected; the limited width reflects the thermodynamics of an entropy-driven system rather than a kinetic limitation.

## Suppression of Autophobic Dewetting via Polymer Architecture

To further investigate the observed nondiffusive interface of L128/d-PLA_43k_, the wetting properties of all four bilayers were examined. For this purpose, a hydrogenated linear PLA with a molecular weight of 42 kg/mol (PLA_42k_) was used as the bottom layer, and either a linear or cyclic PLA (L128, L256, C128, or C256) was placed on top to form PLA_42k_/PLA bilayers, as shown in Figure 5(a). Then, these bilayers were annealed at 80 °C for up to 3 days under vacuum, and their surface morphologies were characterized by optical microscopy.

**Figure 5:**
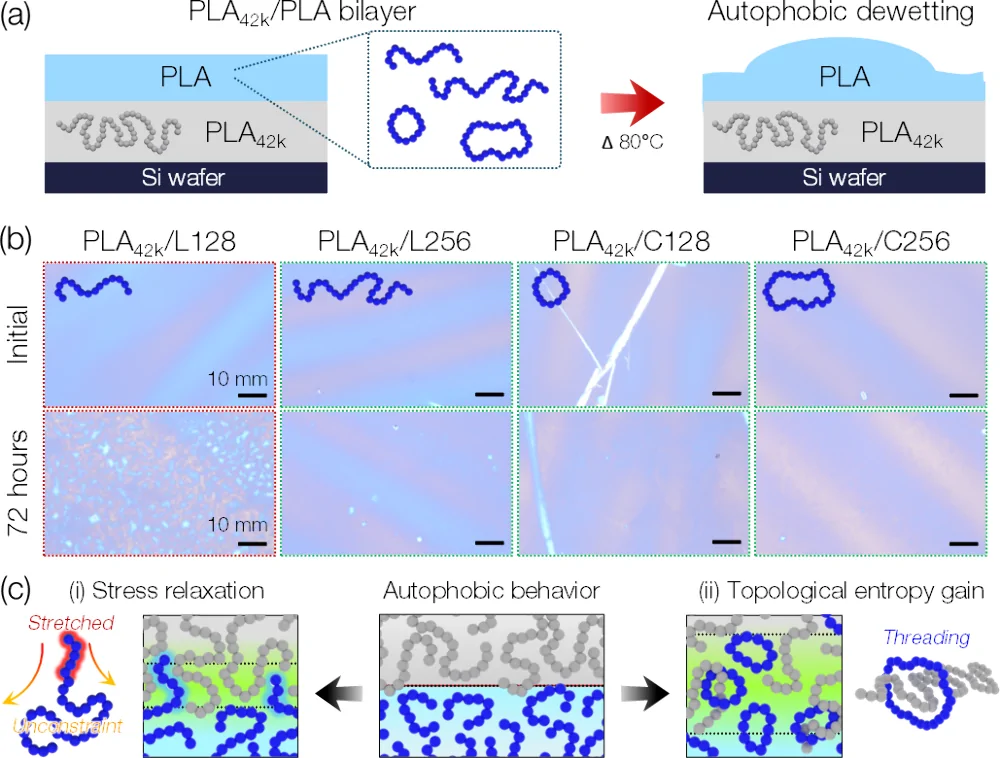
(a) Schematic illustration of the PLA_42k_/PLA bilayer geometry to observe autophobic dewetting. (b) OM images before and after the thermal annealing at 80 °C for 72 h. (c) Schematic illustration of the topological entropy contribution to relaxing autophobicity.

Surface dewetting is observed only at the PLA_42k_/L128 case, after 4 h of annealing (Figure S11), whereas the other combinations remain stable over the entire 3 days, as shown in Figure 5(b). The dewetting found between low and high-molecular-weight linear polymers confirms the incompatibility between the polymers with identical chemistry, a phenomenon often described as autophobic dewetting. The autophobic dewetting was driven by the disparity in molecular weights; thus, increasing the molecular weight of the linear polymer to L256 leads to stable wetting at the PLA_42k_/L256 interface. The details will be discussed in the following section.

Interestingly, the cyclic analogue C128 also exhibits stable wetting on the PLA_42k_ surface despite the same molecular weight as L128. This contrast further highlights the role of polymer architecture in interfacial miscibility. Consistent with the NR results, the dewetting behavior further shows that differences in polymer architecture produce distinct interfacial responses in chemically identical PLA bilayers. Notably, the topology-dependent wetting behavior was also observed in fully hydrogenated bilayers, indicating that the central trend does not originate from H/D isotope substitution used in the NR experiments.

## Discussion

Together, these observations suggest that changes in polymer architecture can shift interfacial miscibility even between polymers of identical chemical composition. Entropic incompatibility, which gives rise to autophobicity, emerges when the S_trans_ gain upon mixing is insufficient to offset the S_conf_ penalty associated with chain penetration across the interface.^30^ This conformational penalty becomes more severe as the molecular weight of the shorter polymer decreases, because a larger fraction of segments experiences stress at the interface.^31^ For a given short polymer, autophobicity is further enhanced as the molecular-weight disparity increases, since penetration into the longer-chain matrix restricts the number of accessible conformations of the short chains.

In our system, the short chain length of PLA amplify this conformational penalty upon mixing. Consequently, the shorter chains, such as L128, experience a stronger entropic penalty, leading to a nondiffusive interface or autophobic dewetting. Increasing the chain length to L256 alleviates this entropic penalty and begins to interpenetrate into the other layer, as longer chains can better relax interfacial stress through their unconstrained segments,^32^ as illustrated in case (i) of Figure 5(c).

Because autophobic dewetting has been reported mainly in brush/melt systems, we further examined whether the same entropy-driven mechanism operates in melt/melt bilayers. The stability of the L256/L128 bilayer (Figure S12­(a)), in contrast to the dewetting observed for PLA_42k_/L128, is consistent with the trend that autophobicity weakens as the molecular weight disparity decreases. This comparison supports that the fundamental origin of autophobicity, namely the S_conf_ penalty associated with interfacial chain penetration, can also manifest in melt/melt systems.

Although L128 shows approximately 1.7 times greater S_trans_ gain than L256, it remains immiscible due to a large loss in ΔS_conf_ incurred during mixing. This relationship can be expressed as ΔS_conf,DP128_ < ΔS_conf,DP256_ < 0. Compatibility improves slightly with increasing molecular weight; however, the interdiffusion remains limited even in the case of L256, with an interdiffusion width of only ∼2 nm (Figure 4(e)), indicating that the conformational penalty remains a barrier.

Converting one of the linear polymers into its cyclic analogue is expected to further reduce the S_conf_ penalty. Since cyclic polymers already possess a significantly lowered S_conf_ due to the ring closure, additional entropy loss can be minimized even when deformed at the interface to be mixed.^33^ In addition, the cyclic/linear system obtains S_topo_ gain through threading, which was absent in linear/linear systems. Specifically, when cyclic and linear polymers mix, segments of the linear chains thread through the cyclic rings and produce an entropic gain upon mixing (case (ii) of Figure 5(c)), as the coexistence of both architectures is entropically more advantageous than that of two cyclic chains. This net gain arises because the linear chains largely retain their unperturbed melt conformations upon threading, whereas the cyclics, relieved of the nonconcatenation constraint imposed by neighboring rings, swell and recover S_conf_ ^11,34,35^ (see Section J in the Supporting Information for further discussion). The positive entropy change due to threading increases the overall compatibility compared to the linear/linear case and can thus be expressed as ΔS_topo, linear_ = 0 < ΔS_topo, cyclic_.

Taking both ΔS_conf_ and ΔS_topo_ into account, the total entropy of mixing follows ΔS_mix_ = ΔS_trans_ + ΔS_conf_ + ΔS_topo_. ΔS_mix_ is the lowest (most negative) for L128 and the highest (most positive) for C256, following the order: ΔS_mix, L128_ < ΔS_mix, L256_ < ΔS_mix, C128_ < ΔS_mix, C256_. This qualitative comparison aligns with our experimental observation and is consistent with simulation predictions previously reported.^10^

Since a direct approach to isolate and quantify the topological entropic contribution has not yet been established, we utilized χ_eff_ as a proxy to compare the contributions of S_conf_ and S_topo_. According to Helfand and Tagami,^31^ the interdiffusion width and χ_eff_ are related as in eq 2, where *b* is a statistical segment length.

$$\chi_{\mathrm{eff}}=\frac{b^{2}}{6\times {\mathrm{interdiffusion width}}^{2}}$$ 2

This equation is strictly valid for equilibrated systems in the strong segregation regime (χN ≫ 1), and our system does not fully satisfy the segregation condition, thus we treat the extracted χ_eff_ as an apparent, semiquantitative parameter, denoted χ_eff_^NR^, rather than an exact χ. Assuming *b* as 0.7 nm based on a commonly used polyolefin reference volume,^36^ the estimated χ_eff_^NR^ values for PLA bilayers are 0.023 for L256/d-PLA_43k_, 0.0042 for C128/d-PLA_43k_, and 0.0033 for C256/d-PLA_43k_ using the interfacial width obtained from Figure 4. For L128/d-PLA_43k_, the interdiffusion width is undetectable, thus, its χ_eff_^NR^ could not be quantified; nonetheless, such an unresolvably narrow interface implies a correspondingly large χ_eff_^NR^, indicating strongly suppressed interdiffusion. As discussed earlier, increasing chain length from L128 to L256 decreases χ_eff_ by reducing the conformational penalty, while changing architecture to C128 also lowers χ_eff_ through topological effect. The calculated χ_eff_ shows that changing architecture is more effective than increasing the chain length.

We further compared the change of χ_eff_^NR^, Δχ, from L256 or C128 to C256 due to topology and chain length. We note that the following analysis is not intended to yield a precise value, but only to gauge the relative magnitude of the two effects. By dividing Δχ_topo_ by Δχ_conf_, we obtained Δχ_topo_/Δχ_conf_ = (χ_eff,C256_ – χ_eff,L256_)/(χ_eff,C256_ – χ_eff,C128_) = −0.0197/–0.0009 ≈ 22. Here, Δχ_topo_ represents the reduction in Δχ_eff_ from changing architecture (linear to cyclic), while Δχ_conf_ reflects the reduction from increasing cyclic chain length. This rough estimation indicates that altering the polymer architecture is more effective in reducing χ_eff_^NR^ than increasing the chain length. For linears, the alleviation by topological entropy is expected to be weaker due to the chain-end contributions. Moreover, the magnitude of Δχ_topo_ (∼10^–2^) is of the same order as the topological χ reported in simulation,^10^ supporting the interpretation that the enhanced broadening at the linear/cyclic interface is driven by topological-entropy-assisted mixing. Although our estimation is simplified and system-specific, it still suggests that the cyclic architecture provides a stronger entropic driving force for increasing compatibility than doubling the chain length alone. More broadly, these findings show that polymer architecture can be used to tune interfacial miscibility without changing chemical identity.

## Conclusions

In this study, we experimentally found that the unique architecture of cyclic polymers greatly influences the entropy-driven incompatibility at interfaces between polymers of identical chemical composition. The interdiffusion width of PLA bilayers was directly examined by NR, and it revealed that cyclic architecture enhanced interfacial miscibility more effectively than increasing molecular weight. Our observation provides the first experimental evidence of interfacial miscibility shift from immiscible to miscible solely due to the topological effect coming from the cyclic architecture, confirming a prediction made over 40 years ago by Cates and Deutsch.^11^

Beyond this specific demonstration, our findings establish molecular topology as a third, independent design axis for controlling interfacial miscibility, complementing the two conventional handles of χ and chain length. Because it operates without altering chemical identity, topology is orthogonal to these established parameters, allowing interfacial behavior to be tuned while the monomer chemistry, and thus the bulk properties, remains unchanged. This is especially compelling now that cyclic analogues of commodity polymers have recently become synthetically accessible. In multilayer films and coextruded structures, where interfacial adhesion dictates mechanical integrity and resistance to delamination and where compatibilization conventionally relies on added block or grafted copolymers, cyclizing one component could enhance layer stability without introducing a chemically distinct compatibilizer.

We recognize that the present work represents an early step, and a full quantitative generalization across polymer chemistries and processing conditions remains to be established. Nevertheless, the capacity of molecular topology to control interfacial thermodynamics independently of chemistry opens substantial room for exploration, and we believe the cyclic architecture holds considerable untapped potential as a tool for interfacial engineering.

## Supplementary Material



## Notes

Materials and methods; Synthesis of discrete linear PLA; Synthesis of discrete cyclic PLA; 1H NMR spectra; Glass transition temperature of bulk PLAs; Molecular-weight dependence of the Tg in cyclic polymers; Determination of NR measurement conditions; Observation of autophobic dewetting between PLAs; Interfacial stability at L128/L256 interface; Threading-induced conformational entropy gain in cyclic chains; Equilibrium nature of the interdiffusion width (PDF)
