# Supplementary material for: Direct Observation of Topologically Enhanced Interfacial Miscibility in Ring–Linear Polymers
Source: ACS Cent Sci. 2026 Sep 3;12(9):1451–7. doi: 10.1021/acscentsci.6c00828 (PMC13614040; doi:10.1021/acscentsci.6c00828)
Supplement: Supplementary file 1 [file oc6c00828_si_001.pdf]

Supporting Information

Direct Observation of Topologically Enhanced Interfacial Miscibility in Ring–Linear Polymers

Seong Eun Kim[1], Dogyun Kim[2], Se Hwa Park[2], Tae Yeon Kong[1], June Hyuk Lee[3], Kyoung Taek Kim[2,*], and So Youn Kim[1,*]

[1]Department of Chemical and Biological Engineering, Institute of Chemical Processes, Seoul National University, Seoul 08826, Republic of Korea

[2]Department of Chemistry, College of Natural Science, Seoul National University, Seoul 08826, Republic of Korea

[3]Neutron Science Division, Korea Atomic Energy Research Institute, Daejeon 34057, Republic of Korea

A. Materials and Methods

Materials

rac-Lactide (Alfa Aesar) was purified by recrystallization from dry toluene prior to use. Benzyl chloride (99%), triethylamine (≥99%), imidazole (99%), *tert*-butyldimethylsilyl chloride (97%), boron trifluoride diethyl etherate, palladium on activated charcoal (10 wt%) and 4-(dimethylamino)pyridine (DMAP) were purchased from Sigma Aldrich and used without further purification. 1-(3-Dimethylaminopropyl)-3-ethylcarbodiimide hydrochloride (EDC•HCl) and 4-pentynoic acid were purchased from Tokyo Chemical Industry and used without purification. Dichloromethane (DCM), dimethylformamide (DMF), and toluene were distilled over CaH_2 under N_2.

Characterization

^{1}H NMR and ^{13}C NMR spectra were recorded by Varian 500 MHz and Agilent 400-MR DD2 Magnetic Resonance System using $CDCl_3$ as solvent. Gel permeation chromatography (GPC) was performed on an Agilent 1260 Infinity equipped with a PL gel 5 µm mixed D column and differential refractive index detectors. THF was used as an eluent with a flow rate of 0.3 mL min^{-1} at 35 °C. A polystyrene standard kit (Agilent Technologies) was used for calibration. Mass spectra of linear and cyclic discrete PLAs were measured on a Bruker Ultraflex III TOF-TOF mass spectrometer equipped with a Nd:YAG laser (355 nm). The instrument was operated in linear and refractive positive ion modes. External calibration was conducted using the following peptides and proteins as reference: Bradykinin fragment 1-7 (757.3997 Da), Angiotensin II (1046.5423 Da), P14R (1533.8582 Da), ACTH fragment 18-39 (2465.1989 Da), Insulin oxidized B chain (3494.6513 Da), Insulin (5735 Da), Cytochrome c (12362 Da), Apomyoglobin (16952 Da), Aldolase (39212 Da) and Albumin (66430 Da)). The ion is $[M+H]^+$ ion. *trans*-2-[3-(4-*tert*-Butylphenyl)-2- methyl-2-propenylidene]malononitrile (DCTB) was used as a matrix.

Glass transition temperature (T_g) measurement

T_g of synthesized PLAs in thin films was characterized using spectroscopic ellipsometry (M-2000V, J.A.Woollam). Before the T_g scan, all the samples were pre-heated at 80°C for 3 hours to remove the residual solvent and then cooled to 10°C at 3 °C/min. T_g is obtained by averaging the last 3 scans among 4 scans, and all the measurements were conducted under vacuum.

PLA bilayer preparation

PLA bilayers consist of hydrogenated (h-PLA) and deuterated Poly(D-lactide-d4) (d-PLA) with d-PLA on h-PLA geometry denoted as h-PLA/d-PLA. To prepare the PLA bilayers with a discrete interface, first, thin films of h-PLA and d-PLA are prepared by spin-coating the solution (1 – 3 wt%, toluene) on the Si wafer. Film thicknesses are 50 nm for h-PLA and 160 nm for d-PLA. Then the d-PLA film is floated onto the water surface by gentle immersion, followed by stamping using h-PLA spin-coated onto the Si wafer. d-PLA has a molecular weight of 42.5 kg/mol and was purchased from Polymer Source, Inc (Product ID #P60220-d4LA, *Đ*=1.33). Information about all PLAs used in this study is summarized in Table S1.

Prior to the NR measurements, the PLA bilayers were stored under high vacuum over 3 days to remove residual solvent. Although no additional pre-heating step was applied, the absence of significant residual solvent was confirmed by the temperature-dependent NR profiles: upon heating, the critical angle shifted systematically toward lower q (Figure S8), consistent with thermal expansion of the film. If a significant amount of residual solvent had been present, its evaporation upon heating would have caused a decrease in film thickness rather than the observed expansion. We therefore conclude that residual solvent, if present, had a negligible effect on the measurements.

Table S1. Information about PLAs used in this study.

Denomination	Topology	Molecular weight, M_n (kg/mol)	Polydispersity, M_w/M_n	Hydrogenated or Deuterated	Tacticity
L128	Linear	9.4	1.0	Hydrogenated	Amorphous
L256	Linear	9.4	1.0	Hydrogenated	Amorphous
C128	Cyclic	18.2	1.0	Hydrogenated	Amorphous
C256	Cyclic	18.2	1.0	Hydrogenated	Amorphous
d-PLA_{43k}	Linear	42.5	1.33	Deuterated	D-form
PLA_{42k}	Linear	42.2		Hydrogenated	D-form

Neutron reflectivity (NR) experiments

To observe the interfacial diffusion, NR experiments are conducted at 80 °C (35°C above the T_g). PLA bilayers are mounted in the vacuum chamber at room temperature, and then the temperature is raised to the target temperature. NR curves were scanned while holding the target temperature.

After the NR measurements, NR curves were fitted using GenX software (version 3.6.11). A two-layer model was constructed consisting of h-PLA (bottom) and d-PLA (top) layers on a SiO_2 substrate. The thickness of each layer was independently determined by spectroscopic ellipsometry prior to NR measurements and used as input values for fitting.

The h-PLA layer thickness was fixed throughout the fitting procedure, as its relatively low SLD contrast contributes minimally to the overall reflectivity profile. The d-PLA layer thickness was set as a fitted parameter to account for thermal expansion upon annealing. The initial SLD values for each layer were calculated from their respective molecular formulas ($C_3H_4O_2$ for h-PLA and $C_3D_4O_2$ for d-PLA) using the NIST neutron scattering length density calculator, yielding 4.610×10^{-6} Å^{-2} for d-PLA and 1.387×10^{-6} Å^{-2} for h-PLA.

The primary fitted parameter was the interfacial roughness between the h-PLA and d-PLA layers, which directly represents the interdiffusion width. Fitting was initiated with an initial roughness value of zero. To verify convergence, fitting was repeated with multiple initial values. Initialization at zero consistently yielded stable and reproducible results, whereas excessively large initial values led to unphysical convergence due to parameter correlation. The goodness of fit was evaluated using the chi-squared figure of merit.

A. Synthesis of discrete linear PLA[1]

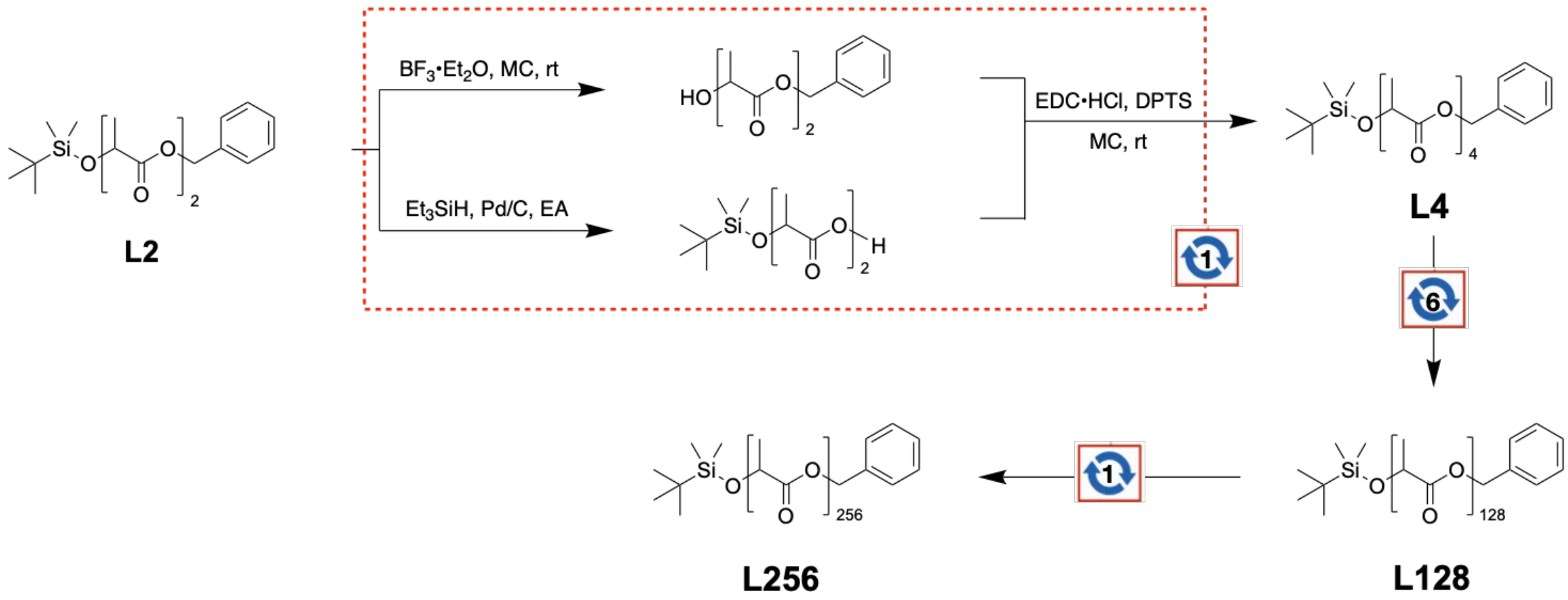

Discrete linear poly(lactic acid)s bearing TBDMS- and benzyl-protected end groups with DPs of 128 (L128), and 256 (L256) were synthesized from the L2 building block by the iterative convergent synthesis according to the general deprotection and coupling procedures described below. The polymers were synthesized through successive chain-length doublings (L2 → L4 → L8 → L16 → L32 → L64 → L128, followed by one additional deprotection/coupling cycle to afford L256). The final products, L128 and L256, were purified by recycling preparative SEC (prep-SEC).

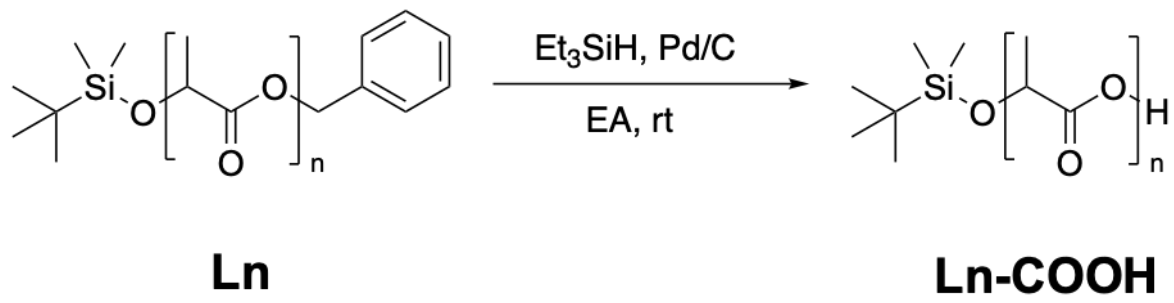

(1) General procedure of debenzylation (Ln to Ln-COOH). Ln was dissolved in ethyl acetate (0.4 M), Pd/C (10 wt%) was added, and the mixture was purged with N_2. Triethylsilane (5 equiv) was added dropwise, and the mixture was stirred at room temperature for 4 h. After completion, the mixture was filtered through Celite, concentrated under reduced pressure, and precipitated into cold hexane; the solid was dried under reduced pressure.

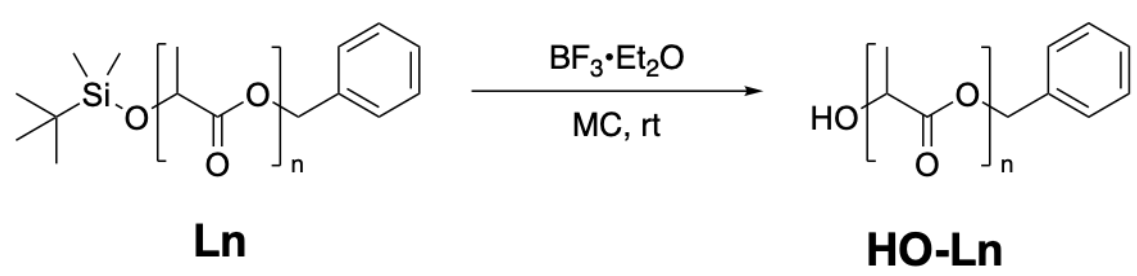

(2) General procedure of desilylation (Ln to HO-Ln). Ln was dissolved in dry DCM (0.4 M) under argon and cooled to 0 °C. $BF_3 \cdot Et_2O$ (4 equiv) was added slowly and the mixture was stirred at room temperature for 2 h. The reaction was quenched with saturated $NaHCO_3$ and washed with brine; the organic layer was dried over $MgSO_4$, filtered, and concentrated. The crude product was purified by automated column chromatography (hexane/EtOAc).

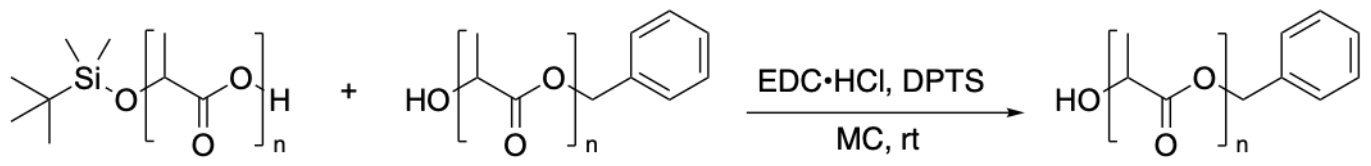

Ln-COOH **HO-Ln** **L2n**

(3) General procedure of coupling. Equimolar Ln-COOH and HO-Ln (obtained by debenzylation and desilylation of Ln, respectively) were dissolved in dry DCM (0.4 M) under argon and cooled to 0 °C. DPTS (0.3 equiv) and EDC·HCl (2 equiv) were added, and the mixture was stirred at room temperature for 12 h. The mixture was washed with water and brine, dried over $MgSO_4$, and concentrated. Products with DP < 64 were purified by automated column chromatography (hexane/EtOAc), whereas products with DP ≥ 64 were purified by recycling prep-SEC.

i) **L4:** Colorless oil (16 g, 95%). ^{1}H NMR (500 MHz, $CDCl_3$): δ 7.40–7.30 (m, 5H, Ph-**H**), 5.25–5.07 (m, 5H, O-C**H**(CH_3)-C=O and Ph-C**H**$_2$-O), 4.41 (q, 1H, SiO-C**H**(CH_3)-C=O), 1.60–1.42 (m, 12H, C**H**$_3$-CH), 0.91 (s, 9H, (C**H**$_3$)$_3$C-Si), 0.10 (dd, 6H, (CH_3)$_3$C-Si(C**H**$_3$)$_2$-O).

ii) **L8:** Colorless oil (11 g, 92%). ^{1}H NMR (500 MHz, $CDCl_3$): δ 7.40–7.30 (m, 5H, Ph-**H**), 5.27–5.07 (m, 9H, O-C**H**(CH_3)-C=O and Ph-C**H**$_2$-O), 4.41 (q, 1H, SiO-C**H**(CH_3)-C=O), 1.60–1.42 (m, 24H, C**H**$_3$-CH), 0.91 (s, 9H, (C**H**$_3$)$_3$C-Si), 0.10 (d, 6H, (CH_3)$_3$C-Si(C**H**$_3$)$_2$-O).

iii) **L16:** Colorless oil. (8.1 g, 90%) ^{1}H NMR (500 MHz, $CDCl_3$): δ 7.40–7.30 (m, 5H, Ph-**H**), 5.26–5.10 (m, 17H, O-C**H**(CH_3)-C=O and Ph-C**H**$_2$-O), 4.39 (q, 1H, SiO-C**H**(CH_3)-C=O), 1.60–1.42 (m, 48H, C**H**$_3$-CH), 0.91 (s, 9H, (C**H**$_3$)$_3$C-Si), 0.10 (s, 3H, (CH_3)$_3$C-Si(C**H**$_3$)$_2$-O), 0.08 (s, 3H, (CH_3)$_3$C-Si(C**H**$_3$)$_2$-O); MS (MALDI-TOF): *m/z* calcd for $C_{61}H_{86}O_{33}$+Na^+ $[M+Na]^+$ 1397.47; found 1397.52.

iv) **L32:** White solid. (5.6 g, 94%) ^{1}H NMR (500 MHz, $CDCl_3$): δ 7.40–7.30 (m, 5H, Ph-**H**), 5.26–5.10 (m, 33H, O-C**H**(CH_3)-C=O and Ph-C**H**$_2$-O), 4.38 (q, 1H, SiO-C**H**(CH_3)-C=O), 1.60–1.42 (m, 96H, C**H**$_3$-CH), 0.91 (s, 9H, (C**H**$_3$)$_3$C-Si), 0.09 (s, 3H, $(CH_3)_3$C-Si(C**H**$_3$)$_2$-O), 0.08 (s, 3H, $(CH_3)_3$C-Si(C**H**$_3$)$_2$-O); MS(MALDI-TOF): *m/z* calcd for $C_{109}H_{150}O_{65}+Na^+$ $[M+Na]^+$ 2549.81; found 2550.34.

v) **L64:** White solid (4.8 g, 93%). ^{1}H NMR (500 MHz, $CDCl_3$): δ 7.40–7.30 (m, 5H, Ph-**H**), 5.27–4.98 (m, 65H, O-C**H**(CH_3)-C=O and Ph-C**H**$_2$-O), 4.38 (q, 1H, SiO-C**H**(CH_3)-C=O), 1.60–1.42 (m, 192H, C**H**$_3$-CH), 0.89 (s, 9H, (C**H**$_3$)$_3$C-Si), 0.09 (s, 3H, $(CH_3)_3$C-Si(C**H**$_3$)$_2$-O), 0.07 (s, 3H, $(CH_3)_3$C-Si(C**H**$_3$)$_2$-O); MS (MALDI-TOF): *m/z* calcd for $C_{205}H_{278}O_{129}+Na^+$ $[M+Na]^+$ 4854.49; found 4855.99.

vi) **L128:** White solid (4.0 g, 86%). ^{1}H NMR (500MHz, $CDCl_3$): δ 7.40–7.30 (m, 5H, Ph-**H**), 5.18 (m, 129H, O-C**H**(CH_3)C=O and PhC**H**$_2$-O), 4.39 (q, 1H, SiO-C**H**(CH_3)C=O), 1.53-1.43 (m, 384H, C**H**$_3$-CH), 0.88 (s, 9H, (C**H**$_3$)$_3$C-Si), 0.10 (dd, 6H, $(CH_3)_3$C-Si(C**H**$_3$)$_2$-O) ppm; MS (MALDI-TOF): molecular *m/z* calcd for $C_{397}H_{534}O_{257}Si+Na^+$ $[M+Na]^+$: 9469.46; found 9469.55.

vii) **L256:** White solid (2.8 g, 72%). ^{1}H NMR (500 MHz, $CDCl_3$): δ 7.40–7.30 (m, 5H, Ph-**H**), 5.18 (m, 257H, O-C**H**(CH_3)C=O and PhC**H**$_2$-O), 4.39 (q, 1H, SiO-C**H**(CH_3)C=O), 1.53-1.43 (m, 768H, C**H**$_3$-CH), 0.88 (s, 9H, (C**H**$_3$)$_3$C-Si), 0.10 (dd, 6H, $(CH_3)_3$C-Si(C**H**$_3$)$_2$-O) ppm; MS (MALDI-TOF): molecular *m/z* calcd for $C_{781}H_{1046}O_{513}Si+Na^+$ $[M+Na]^+$: 18693.52; found 18691.30.

B. Synthesis of discrete cyclic PLA

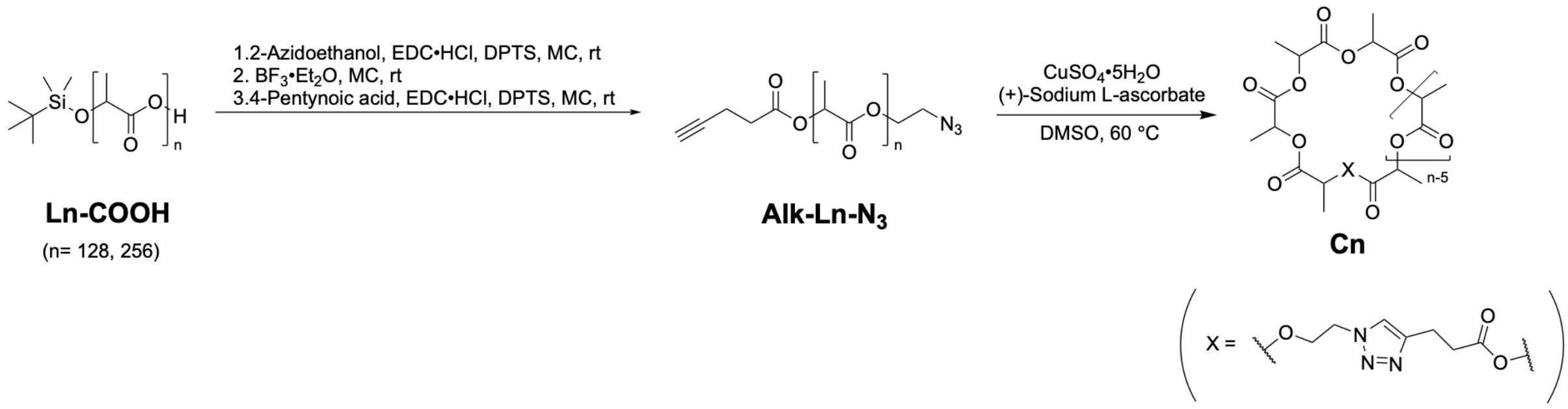

Discrete cyclic PLAs (C128 and C256) were prepared from the corresponding discrete linear precursors, Alk-L128-N_3 and Alk-L256-N_3, by end-group functionalization followed by intramolecular click cyclization. Because the cyclic product has a smaller hydrodynamic volume than its linear precursor of identical molecular weight, the cyclic PLA was isolated by preparative SEC. Detailed synthetic procedures are described below.

(1) Synthesis of Alk-Ln-N$_3$.[1] Alk-Ln-N_3 (n= 128, 256) were prepared from Ln-COOH in three sequential steps. First, Ln-COOH (obtained by debenzylation of Ln) was esterified with 2-azidoethanol (3 equiv) using DPTS (0.3 equiv) and EDC·HCl (2 equiv) in dry DCM (0.4 M) at 0 °C to room temperature for 12 h to give Ln-N_3. The TBDMS group was then removed with BF_3·Et_2O (4 equiv) in dry DCM at 0 °C to room temperature for 2 h to afford HO-Ln-N_3. Finally, HO-Ln-N_3 was esterified with 4-pentynoic acid (3 equiv) using DPTS (0.9 equiv) and EDC·HCl (6 equiv) in dry DCM (0.4 M) at 0 °C to room temperature for 12 h to give Alk-Ln-N_3. After each step, the mixture was washed with water (or, for the desilylation, quenched with saturated $NaHCO_3$) and brine, dried over $MgSO_4$, concentrated, and the product was purified by precipitation into cold diethyl ether.

viii) **Alk-L128-N$_3$:** A white solid; 1H NMR (500MHz, CDCl3): δ 5.20-5.04 (m, 128H, OC**H**(CH_3)-C=O), 4.23 (t, 2H, O-C**H**$_2$-CH_2-N), 3.42 (t, 2H, O-CH_2-C**H**$_2$-N), 2.57 (t, 2H, C=OC**H**$_2$-CH_2-C), 2.45 (t, 2H, C=O-CH_2-C**H**$_2$-C), 1.91 (s, 1H, C**H**-C-CH_2), 1.60-1.42 (m, 384H, C**H**$_3$-CH) ppm; MS (MALDI-TOF): *m/z* calcd for $C_{391}H_{521}NO_{258}$+Na^+ $[M+Na-N_2]^+$: 9386.21.23; found 9387.79.

ix) **Alk-L256-N$_3$:** A white solid; ^{1}H NMR (500MHz, $CDCl_3$): δ 5.20-5.04 (m, 256H, OC**H**(CH_3)-C=O), 4.23 (t, 2H, O-C**H**$_2$-CH_2-N), 3.42 (t, 2H, O-CH_2-C**H**$_2$-N), 2.57 (t,

2H, C=OC**H**$_2$-CH$_2$-C), 2.45 (t, 2H, C=O-CH$_2$-C**H**$_2$-C), 1.91 (s, 1H, C**H**-C-CH$_2$), 1.60-1.42 (m, 768H, C**H**$_3$-CH); MS (MALDI-TOF): *m/z* calcd for $C_{775}H_{1033}NO_{514}$+Na^+ $[M+Na-N_2]^+$: 18610.28; found 18610.97.

(2) **Intramolecular cyclization (C128 and C256)[2].** The cyclization was performed under the following conditions. A mixture of $CuSO_4 \cdot 5H_2O$ (30 equiv) and (+)-sodium L-ascorbate (36 equiv) in DMSO was sparged with argon for 15 min. A degassed DMSO solution of Alk-Ln-N_3 (100 mg) was added dropwise to the copper solution by syringe pump at 60°C at various feed rates (see Table S1 for conditions). After complete addition, the mixture was stirred for a further 4 h. The solution was diluted with DCM, washed with aqueous Na_2EDTA to remove copper salts and then with brine, and concentrated under reduced pressure. The crude product was purified by prep-SEC.

x) **C128:** ^{1}H NMR (500 MHz, $CDCl_3$): δ 7.48 (s, 1H, N-CH-C), 5.26-5.10 (m, 128H, O-CH(CH_3)-C=O), 4.56 (t, 2H, O-CH_2-CH_2-N), 4.51 (t, 2H, O-CH_2-CH_2-N), 3.05 (t, 2H, C=O-CH_2-CH_2-C), 2.78 (t, 2H, C=O-CH_2-CH_2-C), 1.60-1.42 (m, 384H, CH_3-CH) ppm; MS (MALDI-TOF): *m/z* calcd for $C_{391}H_{521}N_3O_{258}$+Na^+ $[M+Na]^+$: 9414.22; found 9414.17.

xi) **C256:** ^{1}H NMR (500 MHz, $CDCl_3$): δ 7.48 (s, 1H, N-CH-C), 5.26-5.10 (m, 256H, O-CH(CH_3)-C=O), 4.56 (t, 2H, O-CH_2-CH_2-N), 4.51 (t, 2H, O-CH_2-CH_2-N), 3.05 (t, 2H, C=O-CH_2-CH_2-C), 2.78 (t, 2H, C=O-CH_2-CH_2-C), 1.60-1.42 (m, 768H, CH_3-CH) ppm; MS (MALDI-TOF): *m/z* calcd for $C_{775}H_{1033}N_3O_{514}$+$Na^+$ $[M+Na]^+$: 18638.29; found 18636.85.

Table S1. Experimental conditions for PLA cyclization

PLA[a]	MW[b] (g/mol)	Feed rate (mL/h)	Concentration (mM)	Solvent (mL)		Concentration (M)	
				Syringe	Batch	$CuSO_4 \cdot 5H_2O$	(+)-sodium L-ascorbate
**Alk-L128-N_3**	9391.23	1	1	10.7	1.06	0.3	0.36
**Alk-L256-N_3**	18615.30	0.5	0.5	10.7	0.54	0.3	0.36

[a]100 mg of Alk-Ln-N_3 was used. [b]Theoretical molecular weights.

C. ^{1}H NMR spectra

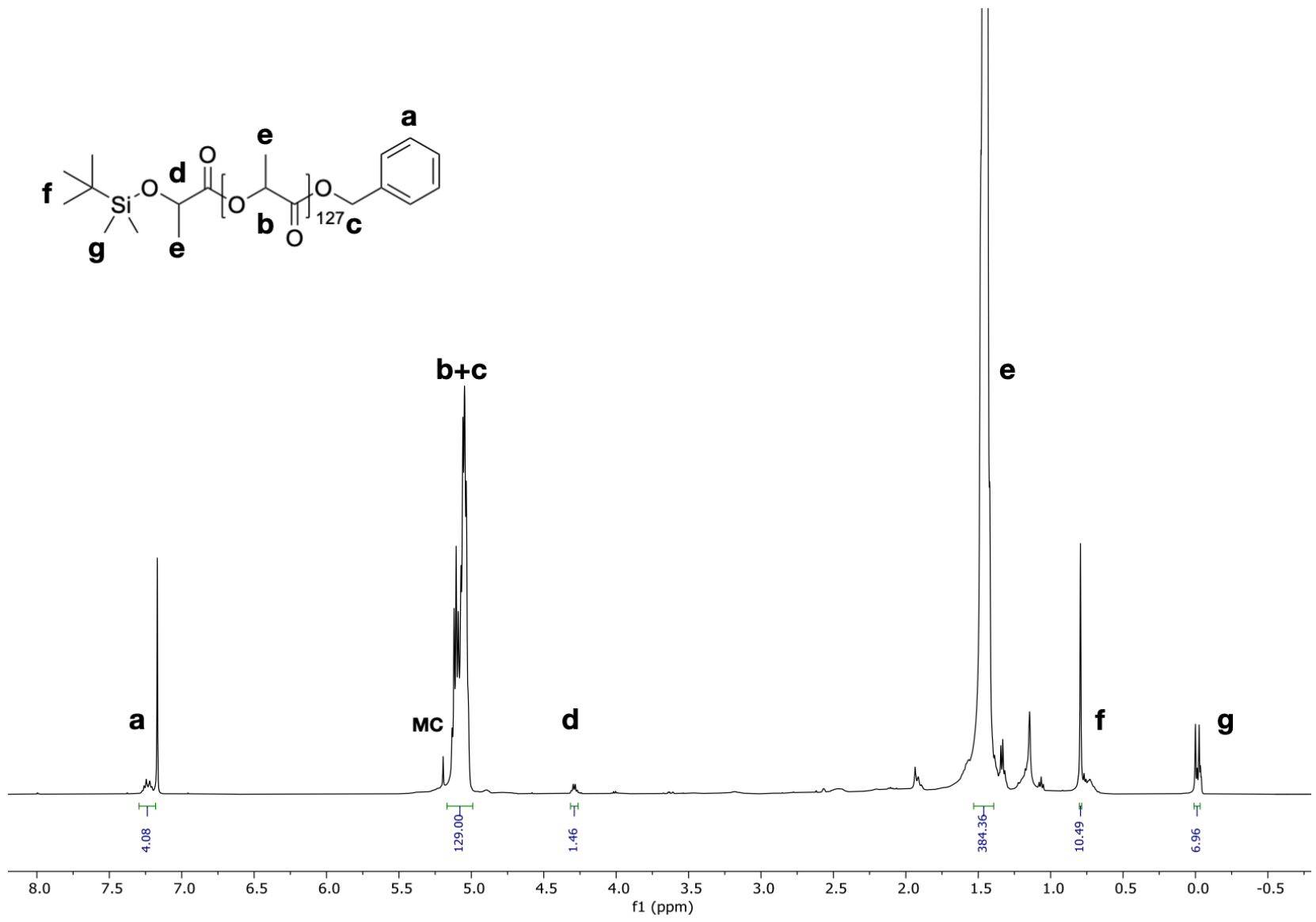

Figure S1. ^{1}H NMR spectrum of L128

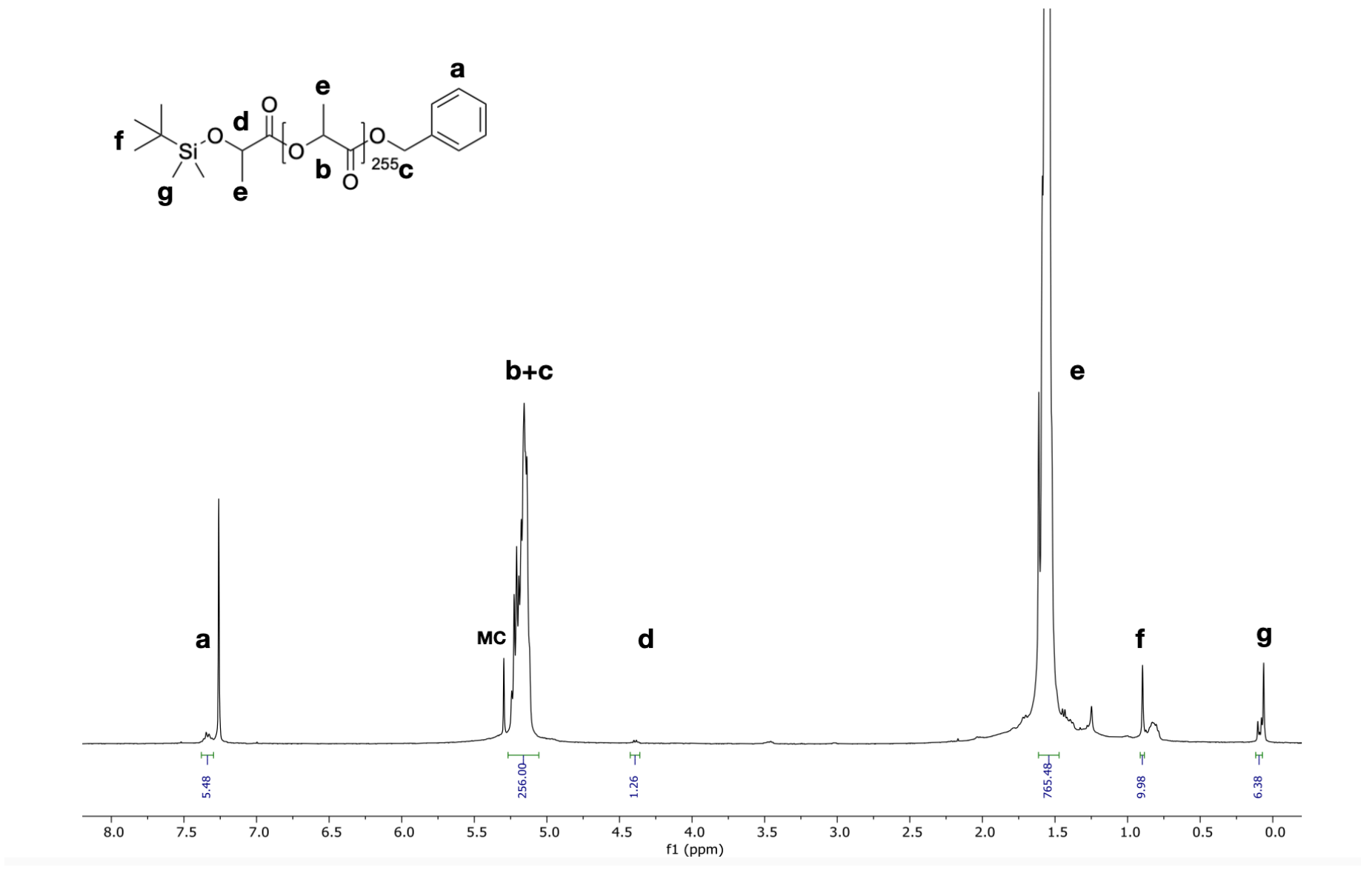

Figure S2. ^{1}H NMR spectrum of L256

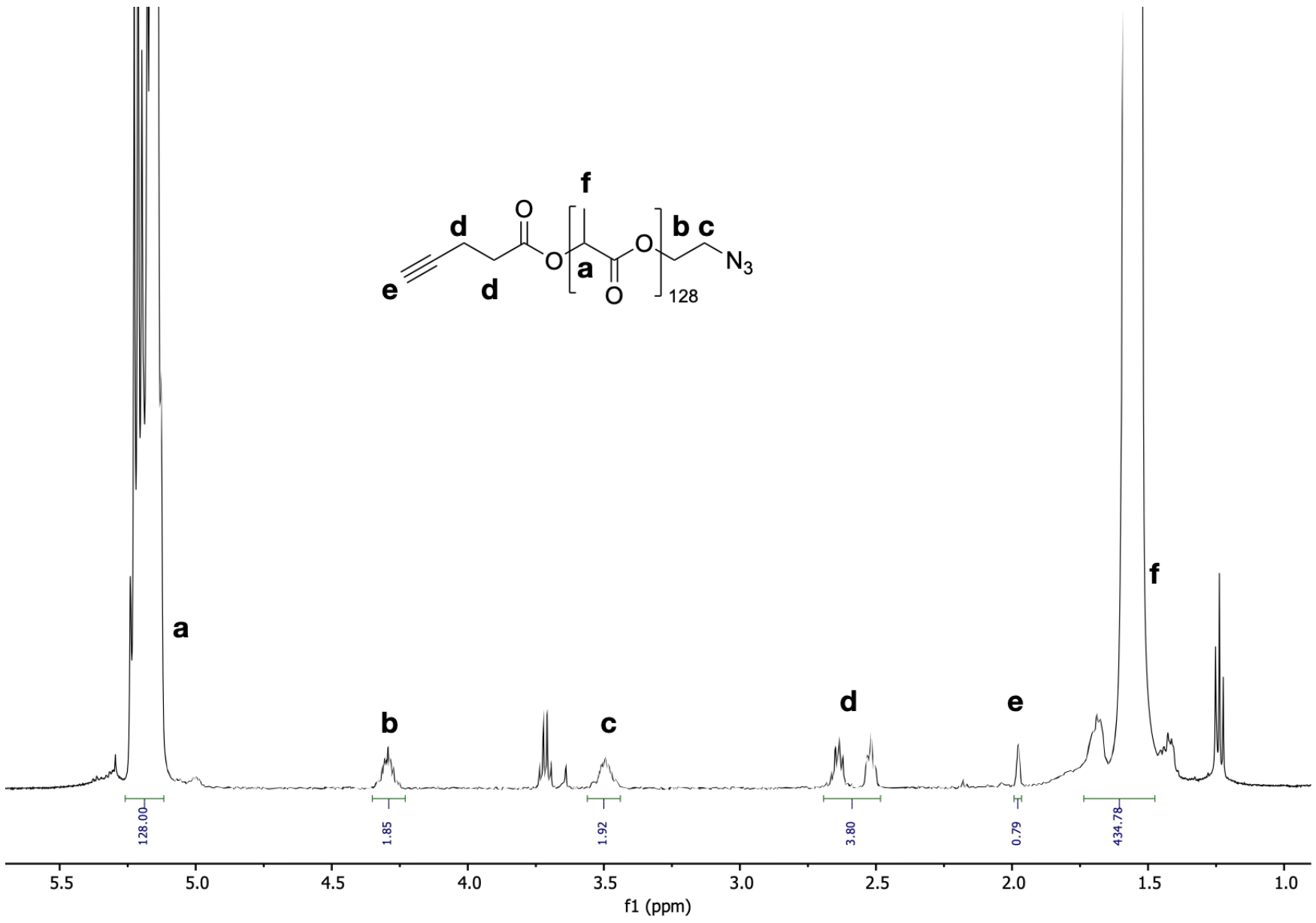

Figure S3. ^{1}H NMR spectrum of Alk-L128-N_3

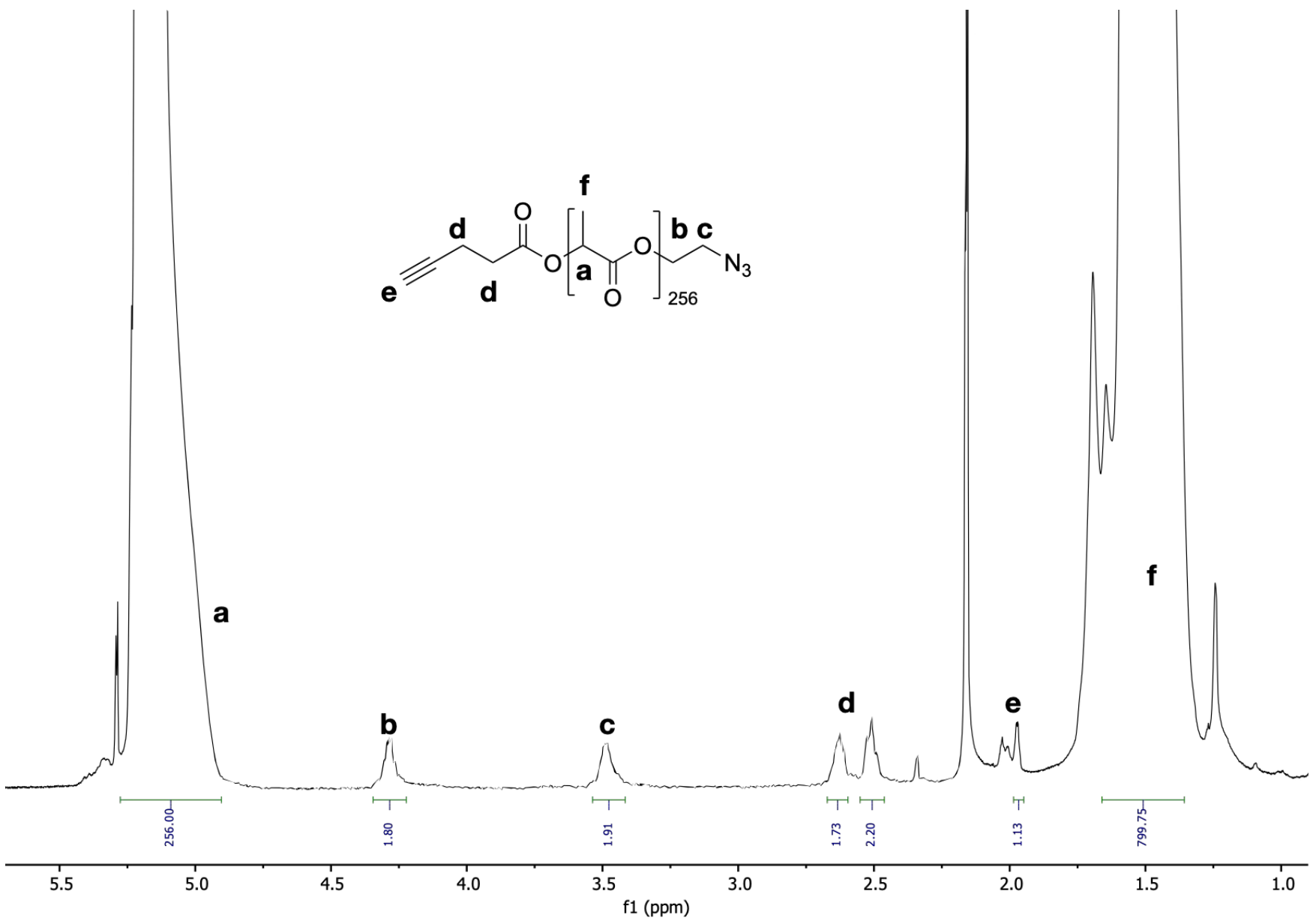

Figure S4. ^{1}H NMR spectrum of Alk-L256-N_3

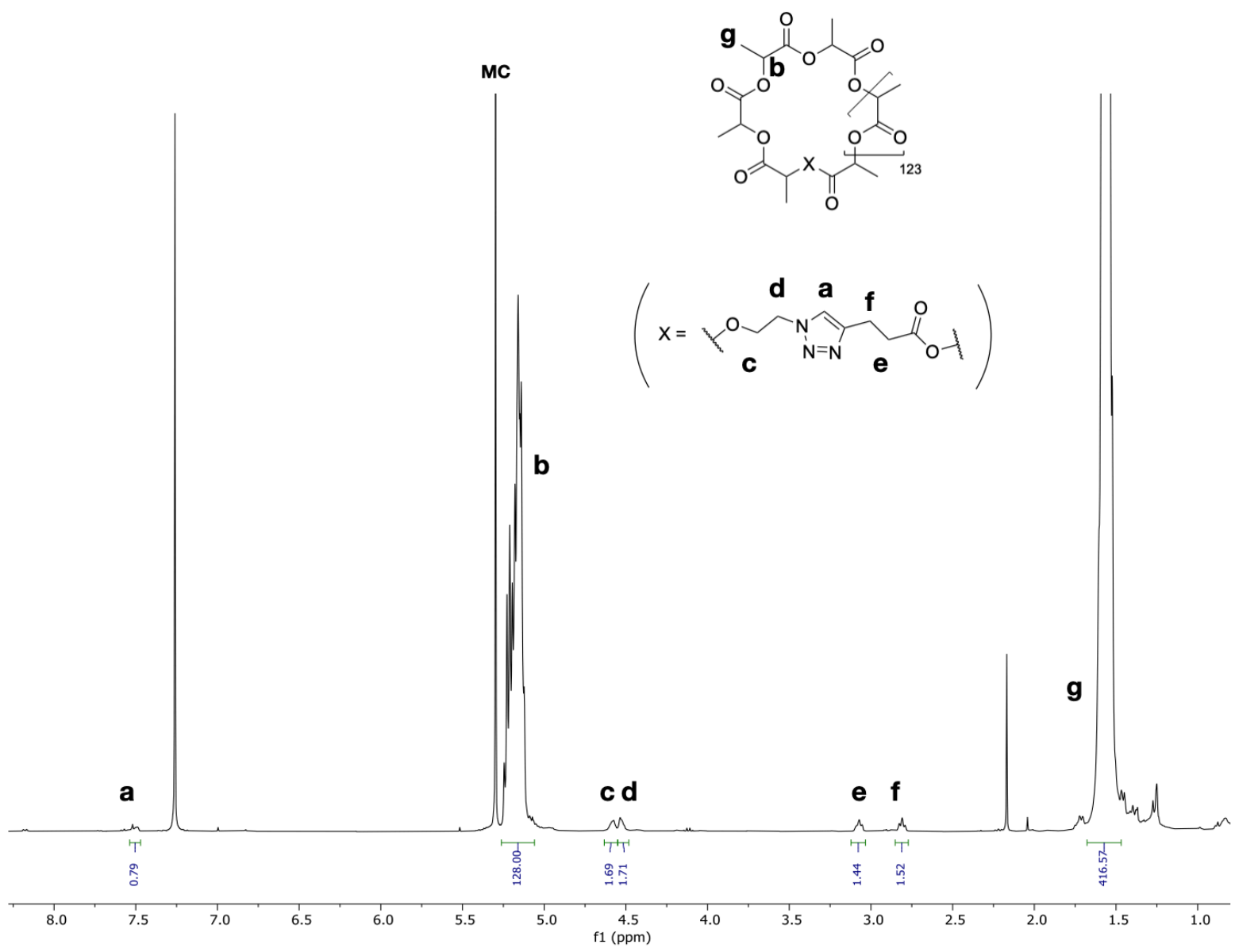

Figure S5. ^{1}H NMR spectrum of C128

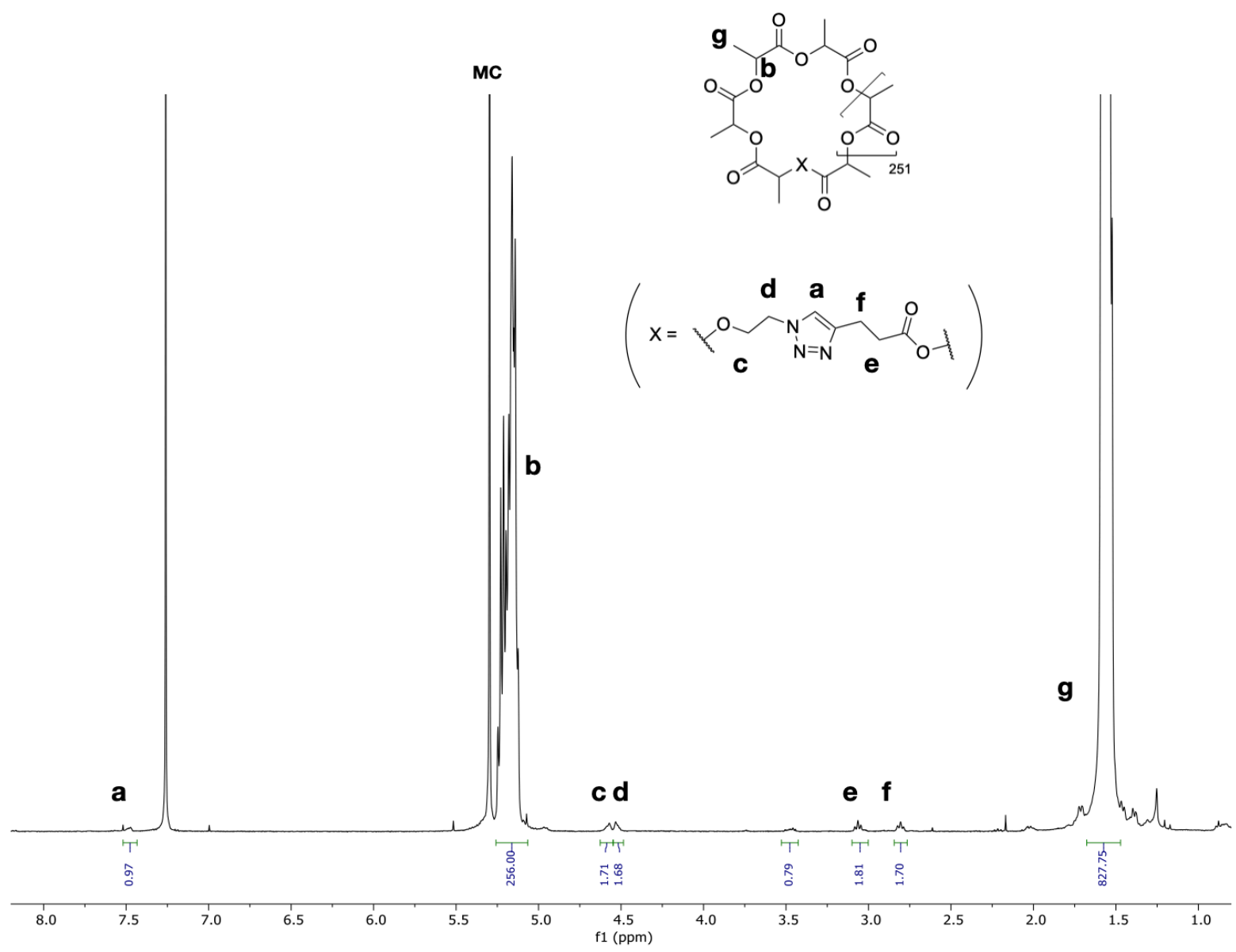

Figure S6. ^{1}H NMR spectrum of C256

D. Glass transition temperature of bulk PLAs

The glass transition temperature (T_g) of the PLAs was measured by differential scanning calorimetry (DSC) to confirm their bulk T_g, as shown in Figure S7. T_g was determined from the intersection between the DSC curve and the midline of the two extrapolated baselines before and after the transition. As shown in Figure S8, all T_g values are slightly higher (by ~3 °C) than those measured by ellipsometry (Figure 2(h)), possibly owing to the difference in measurement method. The trend, however, is identical: T_g increases with molecular weight for the linear analogues, whereas it decreases for the cyclic analogues. This confirms that the observed T_g trend does not originate from measurement error.

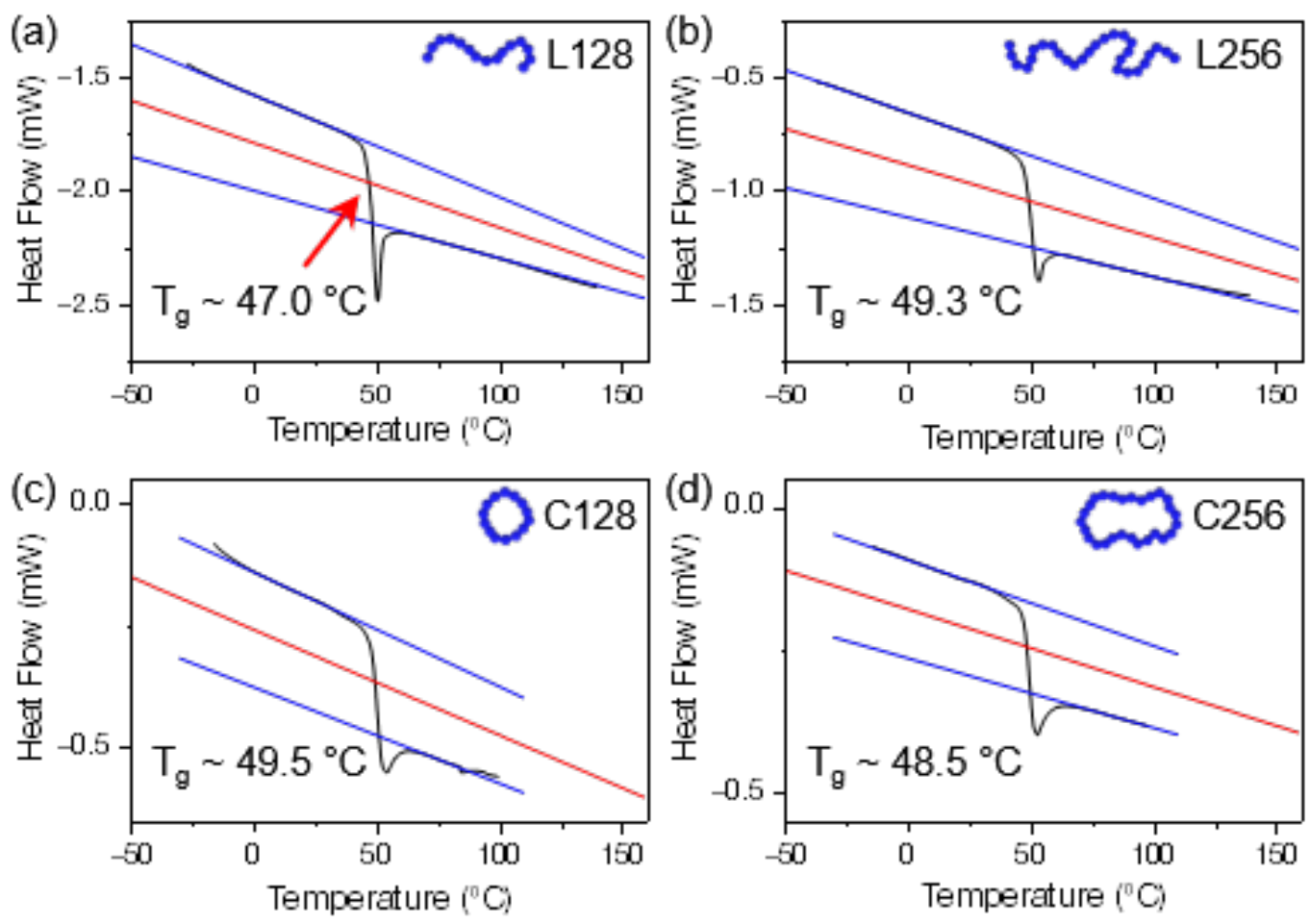

Figure S7. Glass transition of PLAs measured by DSC. Blue lines are the extrapolated baselines before and after the transition, and red lines are the midlines between these baselines. T_g was determined from the intersection of each red midline with the DSC curve.

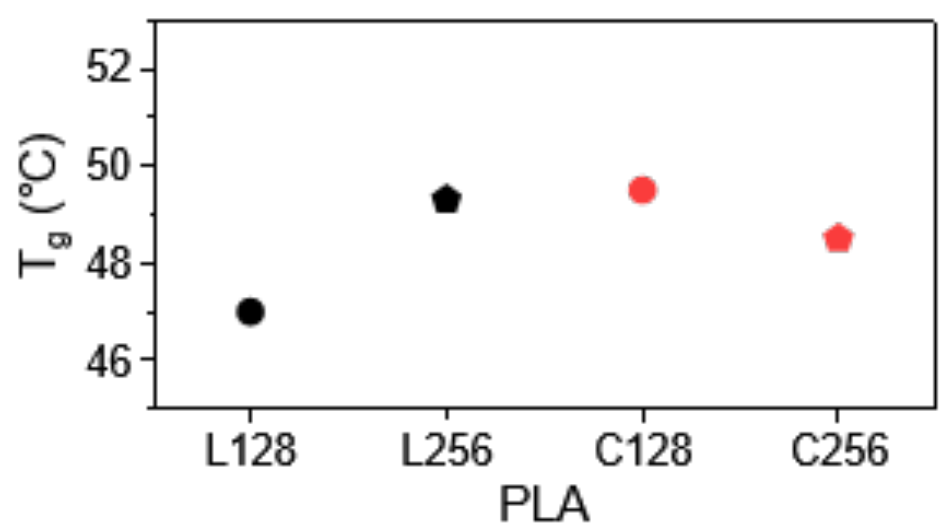

Figure S8. Extracted T_g of PLAs from DSC heating curve

E. Molecular-weight dependence of the T_g in cyclic polymers

The dependence of T_g on molecular weight differs fundamentally between linear and cyclic polymers. For linear chains, T_g decreases as the molecular weight is lowered, a behavior conventionally described by the Fox–Flory relation, $T_g = T_{g,\infty} - K/M_n$, where $T_{g,\infty}$ is T_g in the limit of infinite MW and K is an empirical parameter (related to free volume and chain stiffness) [3]. This decrease originates from chain ends: each end carries excess free volume relative to interior segments, and lowering the molecular weight increases the number density of ends, thereby raising the free volume and depressing T_g.

On the other hand, cyclic polymers have no chain ends, and their molecular-weight dependence has been reported to be qualitatively different; showing an increase, a plateau, or even a decrease in T_g with decreasing molecular weight, depending on the system. Among the reported behaviors, it is now widely attributed to linear contamination when T_g decreases with decreasing molecular weight: even a few percent of residual linear precursor introduces chain ends that lower T_g by several kelvin, an effect well described by the Fox equation[4].

This leaves the distinction between the increasing and plateau-like behaviors, for which no firm consensus has been established. Two different pictures help rationalize these observations. The first framework is from a free-volume standpoint based on chain ends. The ring simply lacks the mechanism that lowers T_g in linear chains; accordingly, this picture predicts a T_g that is nearly independent of molecular weight, or that decreases only slightly. However, it provides no mechanism by which T_g could increase as the molecular weight is lowered. Such an increase is instead predicted by the entropy theory of glass formation, as applied to rings by Di Marzio and Guttman[5].

In this second framework, T_g is the temperature at which the configurational entropy, the number of accessible chain conformations, falls to a critically small value. Because closing a chain into a ring removes conformations that an open chain of the same length could access, a smaller ring is entropically more constrained than a larger one; as the molecular weight is lowered, the configurational entropy is reduced further, and T_g rises. The observation of an increasing T_g with decreasing molecular weight is therefore a distinctive signature of this entropic mechanism, since a chain-end-based free-volume argument cannot account for it.

F. Determination of NR measurement conditions

To determine the appropriate operating temperature, we first scanned the NR profile of the L256/d-PLA bilayer across the temperature range from 30°C to 80°C, as shown in (Figure S1). Below the T_g (~45°C), the NR profiles show no clear oscillations, suggesting the lack of interface sharpening due to limited chain mobility. Oscillations begin to emerge gradually above T_g and become well-defined at 70°C. However, interfacial sharpening and interdiffusion occur simultaneously at high enough temperatures, hindering the identification of diffusion kinetics. Therefore, we selected 80°C as the operating temperature to equilibrate the system quickly, minimizing the kinetic effects during the interdiffusion. At 80°C, the system reaches equilibrium within 20 minutes, with no further changes occurring within a measurable timescale (Figure S2).

Accordingly, the interfacial widths obtained after 15 minutes of annealing were averaged individually for each system to enable comparison.

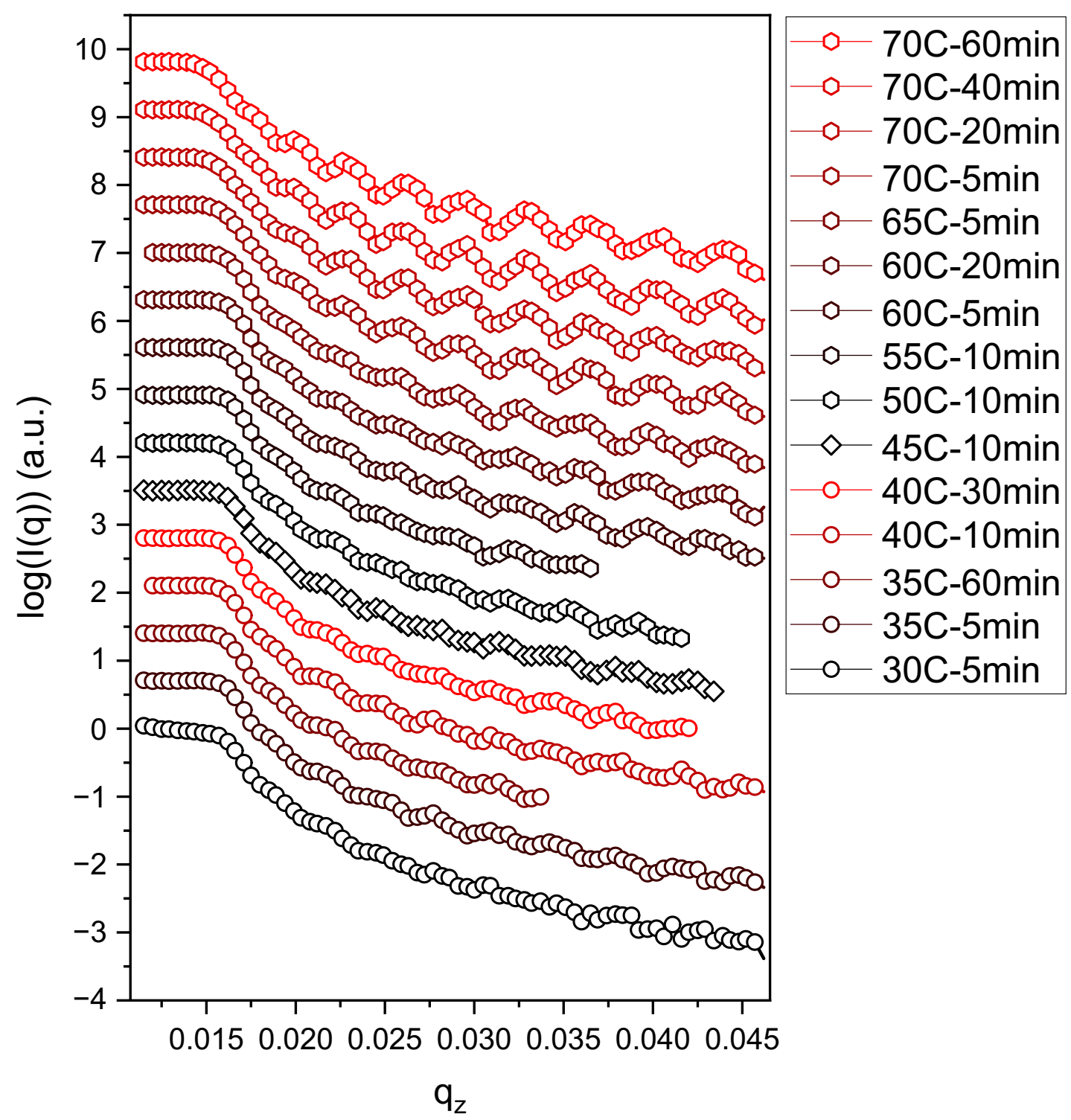

Figure S9. Reflectivity data of L256/d-PLA$_{43k}$ bilayer with increasing temperature

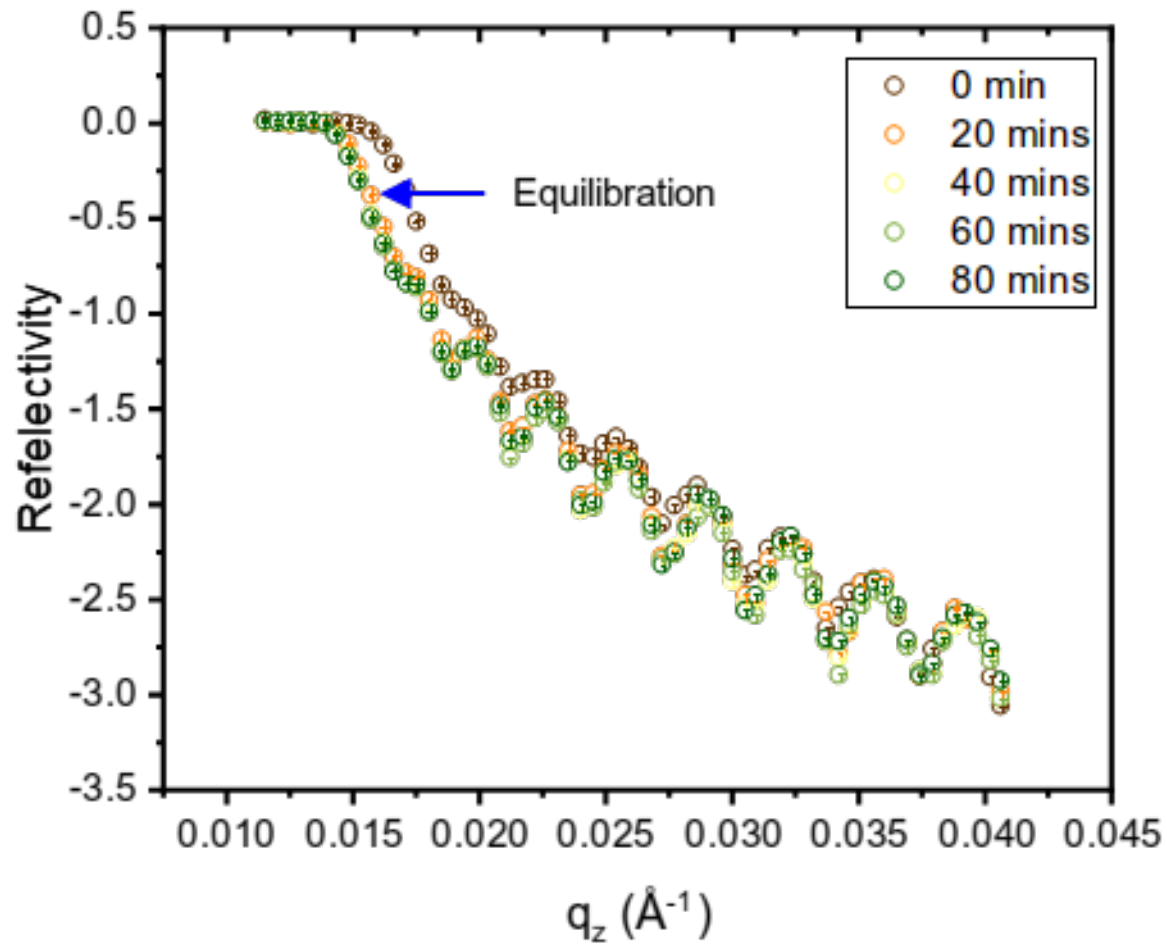

Figure S10. Reflectivity data of L256/d-PLA_{43k} bilayer at fixed 80 °C with increasing annealing time

317 **G. Observation of autophobic dewetting between PLAs**

318 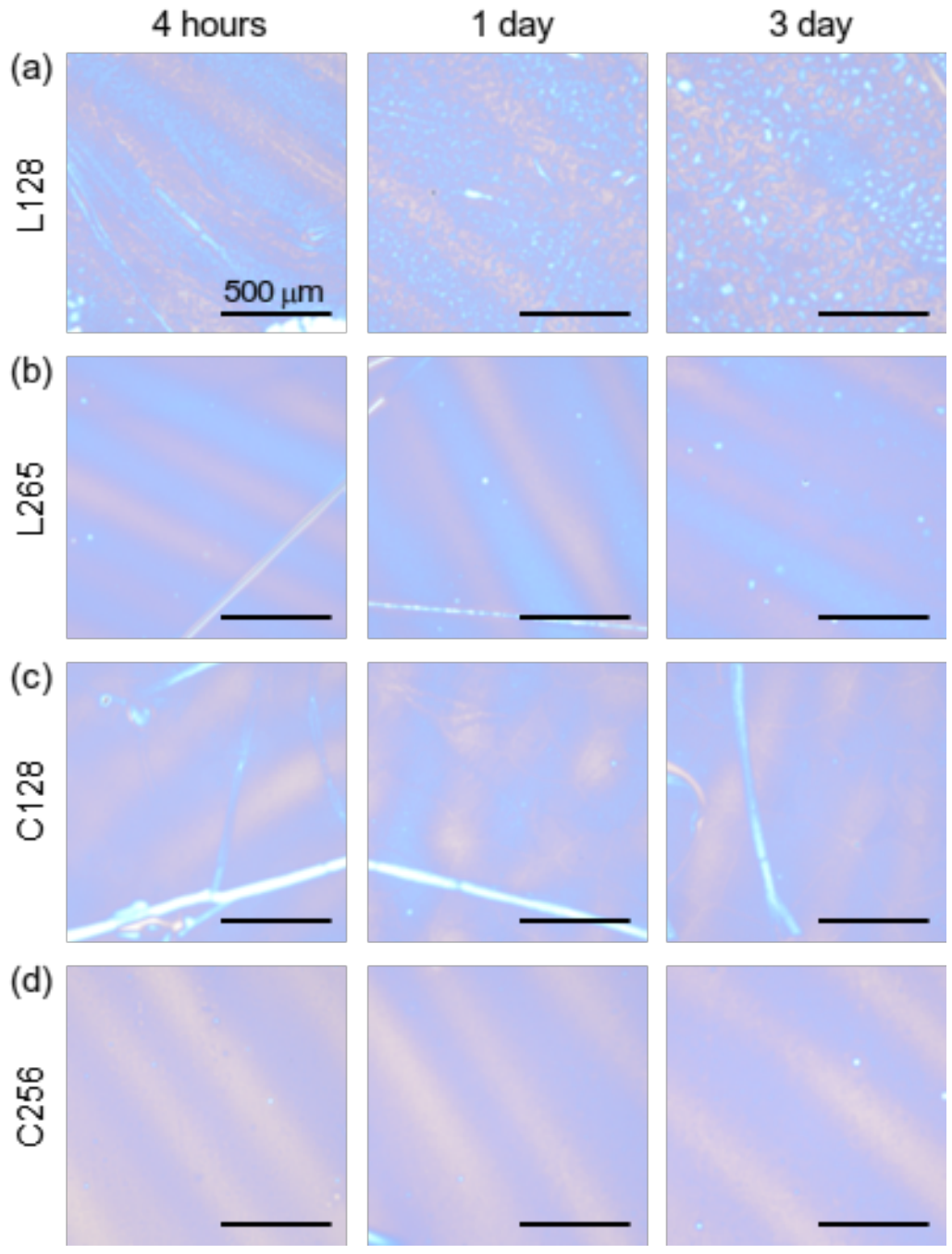

319 Figure S11. Optical microscopy (OM) images of PLA/PLA_{43k} bilayer at fixed 80 °C with
320 increasing annealing time.

321

H. Interfacial stability at L128/L256 interface

When a chemically identical polymer melt attempts to interpenetrate a dense brush, mixing is entropically favored by chemical identity, yet additional stretching and distortion of the brush chains introduce an opposing elastic or conformational free-energy penalty. Autophobicity emerges when this penalty outweighs the entropic gain associated with intermixing. In this sense, the essential mechanism of autophobicity is not the brush architecture itself, but the existence of a strongly constrained interfacial polymer population that resists penetration. Autophobicity should therefore not be viewed as a phenomenon unique to brush/melt systems, but rather as a model case in which conformational constraint at the interface is imposed in a particularly clear and controllable manner.

By extension, the same entropic mechanism may also arise in other polymer/polymer interfaces whenever one component experiences a sufficiently large conformational penalty upon interpenetration. In our melt/melt PLA bilayers, we propose that the short-polymer component plays an analogous role. Although these chains are not grafted, they are expected to bear the larger conformational cost during interfacial mixing, because penetration into the longer-chain matrix requires a greater loss of configurational freedom for the short chains. This effect is likely amplified in PLA, whose relatively rigid backbone increases the penalty associated with chain distortion. From this perspective, the autophobic-like behavior observed in our melt/melt system can be understood as another manifestation of the same underlying entropic mechanism identified in brush/melt autophobicity, except that the conformational constraint here arises from chain-length disparity and backbone rigidity rather than from grafting.

Based on this analogy, we further hypothesized that the dewetting behavior should weaken as the molecular-weight disparity decreases, because autophobicity depends on the P/N ratio, where P and N denote the degrees of polymerization of the melt (long) and brush (short) polymers, respectively. A lower P/N ratio is expected to reduce autophobicity. In other words, replacing PLA_{42k} with L256 while keeping L128 fixed should lead to a more relaxed surface morphology. As shown in Figure S4(a), the L128/L256 bilayer exhibits greater stability than the L128/PLA_{42k} bilayer in Figure S3(a), implying that the dewetting mechanism in our system resembles autophobic dewetting. Although partial surface roughness is observed after prolonged annealing, this roughness originates from the L256 layer itself, as shown in Figure S4(b), rather than from interfacial instability between L128 and L256.

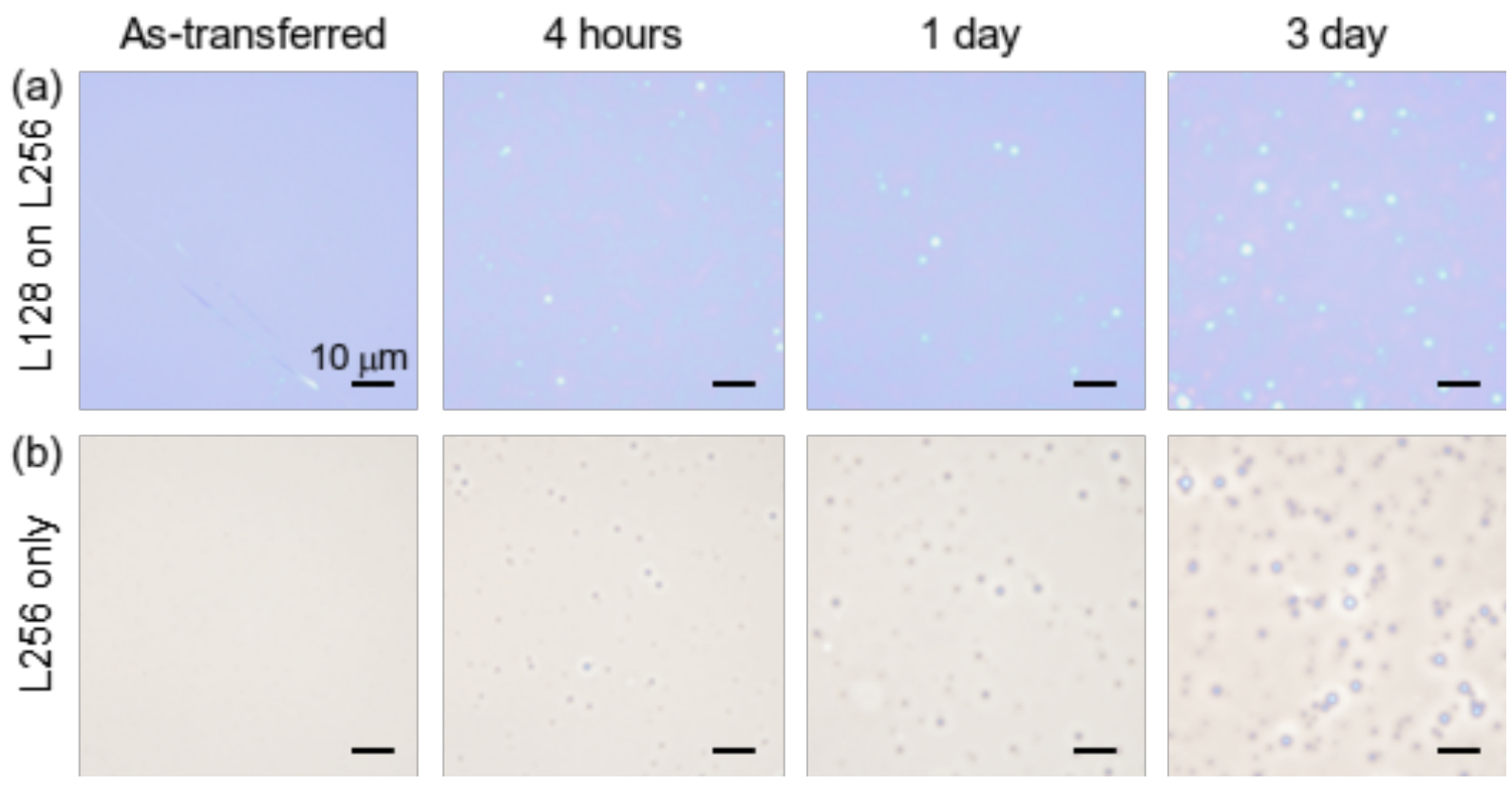

Figure S12. OM image of (a) L128/L256 bilayer and (b) L256 layer after annealing at fixed 80°C.

I. Threading-Induced Conformational Entropy Gain in Cyclic Chains

In a ring-rich (unthreaded, separated) environment, the dominant topological constraint acting on a cyclic chain is non-concatenation with its neighbors. Because two closed loops cannot interpenetrate without becoming permanently concatenated, a cyclic chain must avoid extensive overlap with neighboring rings; the more it expands, the more degrees of freedom it loses to this constraint. As a result, the unthreaded cyclic chain is driven into a compact, segregated conformation that samples only a restricted set of configurations (crumpled globule)-a state of reduced conformational entropy, not unconstrained one as shown in Fig.S13.

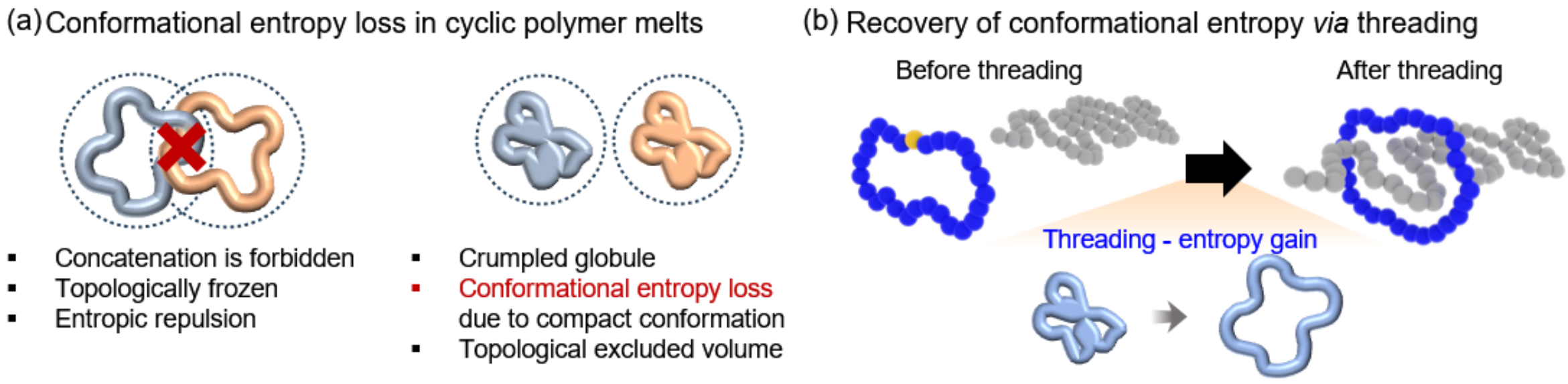

Figure S13. Schematic illustration of (a) configurational entropy loss of each chain in cyclic polymer melts due to non-concatenation rule and (b) conformational entropy gain of cyclic polymer via threading

When the cyclic chain is instead penetrated by linear chains, this penalty is lifted. Threading by a linear chain does not constitute a topologically invariant concatenation: a linear chain and a ring are always separable, so the cyclic chain has no topological reason to avoid such penetration. Released from the non-concatenation compression, the cyclic chain is free to swell and to explore a substantially larger conformational ensemble. This is precisely the classical result of Cates and Deutsch, who showed that a ring in a melt of chemically identical linear chains should be swollen and that the corresponding ring-linear interaction parameter is negative.

Consequently, the threaded cyclic conformation possesses higher conformational entropy than the unthreaded, segregated one: the entropy suppressed by non-concatenation in the unthreaded state is recovered, and can be further enhanced toward a swollen state, upon threading. It is this gain in the cyclic chain's own conformational entropy that constitutes the topological entropy gain (S_{topo}) and provides the thermodynamic driving force for the broader interdiffusion we observe at the cyclic/linear interface.

The linear chains lose their conformational entropy during threading; however, this loss is negligible compared to the topological entropy gain. The key reason is that a linear chain possesses free ends. Unlike a ring, whose accessible conformations are governed by the non-concatenation constraint with its neighbors, a linear chain renews its configurations through end-controlled motion (reptation) and is therefore largely insensitive to whether its neighbors are rings or other linear chains. Replacing a linear neighbor with a cyclic one does not alter the topological constraints acting on the linear chain, so its conformational statistics, and hence its conformational entropy, remain essentially unchanged upon threading.

This expectation is directly supported by simulations of ring–linear blends. Using a scaling model together with bond-fluctuation Monte Carlo simulations, Iyer, Lele, and Shanbhag showed that the radius of gyration of the linear component is independent of blend composition across the entire range from pure linear to pure ring, remaining equal to that of a coil in a pure linear melt [6]. They explicitly attribute this to the fact that the constraints imposed on a linear chain do not change when a linear neighbor is replaced by a cyclic molecule. Subramanian and Shanbhag reached the same conclusion from a topological analysis: both the primitive-path length and the average number of entanglements of the linear chains were found to be independent of blend composition, in sharp contrast to the rings, whose values increased markedly with the linear fraction [7].

Taken together, these results establish that, from the linear chain's perspective, threading is not accompanied by any appreciable reduction in conformational entropy: the linear chain retains its unperturbed, melt-like (Gaussian) dimensions and its characteristic entanglement state regardless of the surrounding architecture.

J. Equilibrium nature of the interdiffusion width

A central assumption in our analysis is that the interdiffusion widths extracted from NR correspond to equilibrium interfacial widths, rather than transient values reflecting incomplete interdiffusion. This distinction matters because the Helfand–Tagami relation used to extract χ_{eff} (Equation 2 in the main text) is strictly valid only for equilibrated interfaces; if the measured widths were kinetically limited, that is, still evolving toward a broader equilibrium value but arrested by slow chain dynamics, the extracted χ_{eff} would not carry thermodynamic meaning. We therefore examined whether the reported widths represent equilibrium states, primarily on the basis of the time-independence of the width under prolonged annealing.

The interfacial width is independent of annealing time. For every bilayer (L128/d-PLA_{43k}, L256/d-PLA_{43k}, C128/d-PLA_{43k}, and C256/d-PLA_{43k}), we tracked the interfacial width as a function of annealing time at fixed 80 °C. In every case, the width increases initially and then reaches a plateau, beyond which it does not change with further annealing (Figure S10 and S14). All widths reported in the main text were averaged values within this time-independent regime. If the interface were still relaxing toward a broader equilibrium configuration, the width would continue to grow with annealing time; a stationary width is what one expects when the system has reached the balance point at which there is no further thermodynamic driving force to broaden the interface. Importantly, this behavior was confirmed for all bilayers, including both cyclic/linear systems, so the equilibrium assignment applies uniformly across the systems compared in this work.

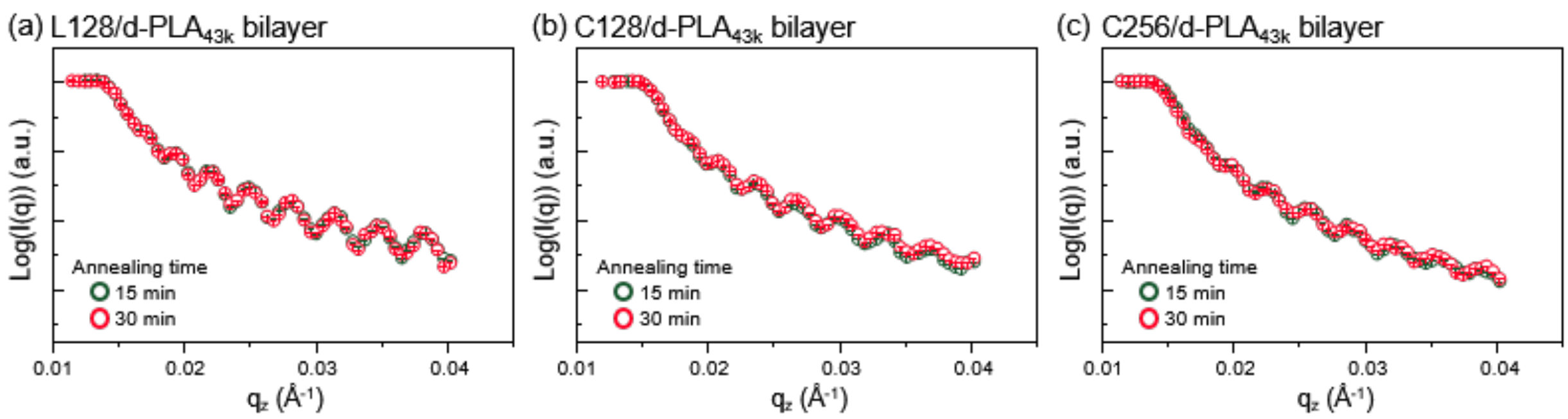

Figure S14. Time-dependent NR curves of (a) L128/dPLA_{43k}, (b) C128/dPLA_{43k}, and (c) C256/dPLA_{43k} bilayer at 80°C

(1) Koo, M. B.; Lee, S. W.; Lee, J. M.; Kim, K. T. Iterative Convergent Synthesis of Large Cyclic Polymers and Block Copolymers with Discrete Molecular Weights. *J. Am. Chem. Soc.* **2020**, 142 (33), 14028-14032.

(2) Noh, J.; Koo, M. B.; Jung, J.; Peterson, G. I.; Kim, K. T.; Choi, T.-L. Monodisperse Cyclic Polymer Mechanochemistry: Scission Kinetics and the Dynamic Memory Effect with Ultrasonication and Ball-Mill Grinding. *J. Am. Chem. Soc.* **2023**, 145 (33), 18432-18438.

(3) Fox, T. G.; Flory, P. J. Second-Order Transition Temperatures and Related Properties of Polystyrene. I. Influence of Molecular Weight. *Journal of Applied Physics* **1950**, 21 (6), 581-591.

(4) Gao, L.; Oh, J.; Tu, Y.; Chang, T.; Li, C. Y. Glass transition temperature of cyclic polystyrene and the linear counterpart contamination effect. *Polymer* **2019**, 170 198-203.

(5) Di Marzio, E. A.; Guttman, C. M. The glass temperature of polymer rings. *Macromolecules* **1987**, 20 (6), 1403-1407.

(6) Iyer, B. V. S.; Lele, A. K.; Shanbhag, S. What Is the Size of a Ring Polymer in a Ring−Linear Blend? *Macromolecules* **2007**, 40 (16), 5995-6000.

(7) Subramanian, G.; Shanbhag, S. Conformational properties of blends of cyclic and linear polymer melts. *Physical Review E* **2008**, 77 (1), 011801.
